# Solid‐State NMR Reveals Reorganization of the *Aspergillus fumigatus* Cell Wall Due to a Host‐Defence Peptide

**DOI:** 10.1002/anie.202509012

**Published:** 2025-07-16

**Authors:** Ajit Kumar Bishoyi, Jacq van Neer, Salima Bahri, Sophie Lorenz, Hans de Cock, Marc Baldus

**Affiliations:** ^1^ NMR Spectroscopy Bijvoet Center for Biomolecular Research Utrecht University Padualaan 8 Utrecht 3584 CH The Netherlands; ^2^ Microbiology Department of Biology Utrecht University Padualaan 8 Utrecht 3584 CH The Netherlands

**Keywords:** Antifungal drug, CATH‐2, Fungal cell wall, ssNMR

## Abstract

The limited availability of antifungal drug treatments and the rising issue of drug resistance highlight the urgent need for new antifungal drugs to combat drug‐resistant *Aspergillus fumigatus*. The host‐defence peptide cathelicidin‐2 has demonstrated a significant inhibitory effect on azole‐resistant *Aspergillus fumigatus* but its mechanism of action remains elusive. We applied a tailored set ^1^H and ^13^C detected solid state nuclear magnetic resonance experiments to elucidate the cell wall composition of *Aspergillus fumigatus* and to shed light on the mechanism of action of the peptide within the cell wall. Our results revealed that presence of the peptide affects galactosaminogalactan, an important component involved in the pathogenesis of invasive aspergillosis, as well as other specific polysaccharides and amino acids within the mobile cell wall domain. At longer exposure times, the peptide also influences the rigid cell wall domains by enhancing water penetration into the hydrophobic rigid cell wall domain. The findings reveal how the peptide can reach the plasma membrane and may aid the design of novel antifungal drugs with enhanced efficacy.

## Graphical abstract

Understanding the mode of action is critical to develop new antifungal drugs. Solid‐state NMR was used to study the reorganization of the *Aspergillus fumigatus* cell wall by a host‐defence peptide. We identified specific polysaccharides and amino acids that are affected by the peptide. Our studies also suggest a mechanism on how the peptide can reach the plasma membrane, which may aid the design of novel antifungal drugs with enhanced efficacy.

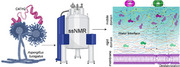

## Introduction

Fungal infections in humans and animals are currently treated with a relatively limited set of antifungal drugs.^[^ ^1^, ^2^, ^3^ ^]^ However, resistance to antifungal drugs is increasing with an unprecedented rate, amongst others due to their extensive use in the environment outside medical care facilities.^[^ ^4^, ^5^, ^6^ ^]^ Consequently, extended exposure of humans and animals to resistant pathogens is expected to lead to a rise in incurable infections. Resistance to antifungal drugs is frequently reported for important fungal pathogens.^[^ ^7^, ^8^ ^]^ Fungal infections are treated with different antifungal drugs like azoles, echinocandins, or amphotericin which have their target in the cytosol or at the plasma membrane. In both cases, such antifungal compounds need to pass the fungal cell wall which may serve as interaction site and affect their ability to reach the plasma membrane or intracellular targets. Solid‐state nuclear magnetic resonance (ssNMR) has proven to be a powerful method to study the architecture of the biomolecular and macromolecular systems, including fungal cell walls^[^ ^9^, ^10^, ^11^, ^12^, ^13^, ^14^, ^15^ ^]^ as well as antifungal peptides.^[^ ^16^, ^17^, ^18^ ^]^ However, atomic‐level insight into how antifungal peptides interact with the cell wall has been limited. In the following, we present a ssNMR approach to reveal the influence of an antimicrobial peptide upon the fungal cell wall in a time‐dependent manner.

Antimicrobial peptides are generally regarded as membrane destabilizing compounds which can be active at the plasma membrane or even intracellularly.^[^ ^19^ ^]^ Previously, it was shown that histatin 5 targets mitochondria in the yeast *Candida albicans (C. albicans)* ^[^ ^20^ ^]^ as well as the fungal vacuole.^[^ ^21^ ^]^ In contrast, two peptides belonging to the cathelicidin family, the only human variant LL‐37 and chicken variant cathelicidin‐2 (CATH‐2), were shown to permeabilize the plasma membrane but also had intracellular targets in *C. albicans*.^[^ ^21^ ^]^ Previously, some of us demonstrated that host‐defence peptides (HDPs) of the cathelicidin family also showed strong inhibition of (azole‐resistant) *Aspergillus fumigatus* (*A. fumigatus*) strains and, in addition, to a large set of clinical relevant fungal species.^[^ ^22^ ^]^ The genus *Aspergillus* contains over 400 different fungal species^[^ ^23^ ^]^ that are amongst the most common moulds known, of which a few are able to cause disease in animals and humans. *A. fumigatus* is the one to cause the most detrimental diseases, such as invasive aspergillosis. *A. fumigatus* is a ubiquitous filamentous fungus that can form small hydrophobic conidia with a size of 2–3 µms in diameter which disperse easily in the air.^[^ ^24^, ^25^ ^]^ It is estimated that humans inhale over hundreds of these conidia per day.^[^ ^26^ ^]^

Cathelicidins are HDPs that are naturally produced during an immune response by a variety of immune cells.^[^ ^27^ ^]^ CATH‐2 is a positively charged chicken derived cathelicidin, a small peptide comprising 26 amino acids (charge 8+) and showed the most promising results regarding the inhibition of *A. fumigatus* strains.^[^ ^28^ ^]^ While Alphafold 2.0^[^ ^29^ ^]^ predicts an N terminal α helix and a disordered C‐terminus for CATH‐2, earlier NMR studies on a CATH‐2 variant reported an helix‐hinge‐helix structure^[^ ^30^ ^]^ in organic solvent (Figure S1).

Thus far, the mode of action of the peptide remains unclear nor is it known which molecular components of the fungal cell wall are affected. Previous studies have already examined the composition and the architecture of *A. fumigatus* cell wall using ^13^C‐detected solid‐state NMR.^[^ ^12^, ^13^, ^31^ ^]^ The rigid domain of *A. fumigatus* (strains CEA17ΔakuBKU80 and Af293) is comprised of a hydrophobic complex of α‐1,3‐glucan and chitin, which is contained in a matrix of β‐glucans. The mobile domain primarily consists of galactomannan (GM), galactosaminogalactan (GAG), β‐glucans and proteins.^[^ ^12^, ^13^ ^]^ Furthermore, ssNMR studies revealed the compositional changes during different life cycle stages of *A. fumigatus*.^[^ ^31^ ^]^ In parallel, our groups have previously used ssNMR to study the composition of the basidiomycete *Schizophyllum commune* (*S. commune*) cell wall^[^ ^32^, ^33^ ^]^ and its interaction with metal ions and CATH‐2.^[^ ^33^, ^34^ ^]^ In the latter case, we isolated cell wall material and developed proton‐detected ssNMR experiments^[^ ^33^, ^35^ ^]^ that allowed us to resolve changes in the cell wall in an additional spectral dimension without the need of isotope labeling^[^ ^36^ ^]^ and to dissect the relative polysaccharide abundance.^[^ ^11^, ^33^ ^]^

Here, we combined ^13^C‐ and ^1^H‐detected ssNMR methods to characterize the cell wall of *A. fumigatus* and investigate its interaction with CATH‐2. For our experiments, we devised a dedicated experimental setup to probe peptide binding to the infectious *A. fumigatus* in an NMR laboratory setting. Using a combination of ^13^C‐ and ^1^H‐detected ssNMR experiments, we determined the molecular composition of rigid and dynamic cell wall components of *A. fumigatus* in fungal mycelia and compared our data to previous reports using isolated cell wall components. Subsequently, we used such methods to probe the cell wall effect of CATH‐2 for two different incubation times. These studies revealed molecular species that are influenced by the inhibition of the peptide. Interestingly, longer incubation times also affected the rigid domain of the cell wall and modified the water‐exposed interface between dynamic and rigid cell wall components. Using a novel ssNMR pulse scheme, we found that these interfaces are rich in β‐ glucan and α‐ glucan, comprise chitin and chitosan and exhibit a high degree of monomorphism.

## Results

### Functional Characterization of CATH‐2 and ssNMR Sample Preparation

CATH‐2 was produced by China peptides (see Supporting Information Materials & Methods), which maintained a stable structure at 37 °C as revealed by ^1^H solution‐state NMR (Figure S2). To further understand whether CATH‐2 inhibits the growth of the *A. fumigatus*, we monitored *A. fumigatus* growth over 24 h at concentrations ranging from 0 to 5 µM of CATH‐2 using the oCelloScope. 5 µM of CATH‐2 effectively suppressed the early stages of the growth such as spore swelling (Figure S3a) and germ tube formation (Figure S3b), that are very crucial stages in its development. Based on these results, an optimum concentration of 5 µM CATH‐2 was chosen for the ssNMR experiments to investigate which molecular species are affected by peptide inhibition of the *A. fumigatus* cell wall.

**Figure 1:**
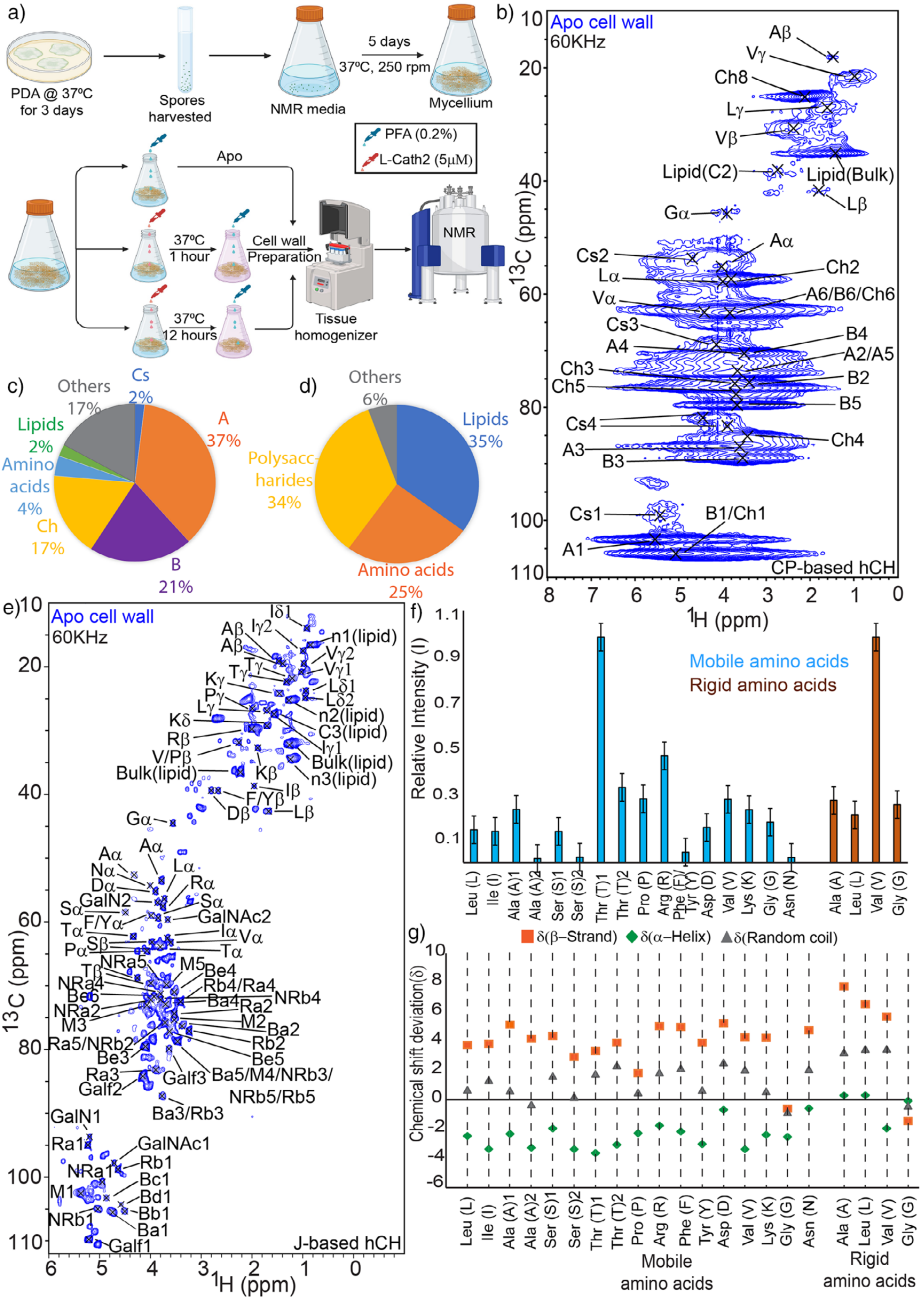
Characterization of both the rigid and mobile domains. a) Schematic diagram of the sample preparation of *A. fumigatus* cell wall for ssNMR (see also Supporting Information). b) ^1^H‐detected CP‐ based 2D hCH spectrum; c) Relative quantification of the compounds in the rigid domain based on the CP‐ based 2D hCH spectrum. A = α‐(1,3)‐glucan, B = β‐(1,3)‐glucan, Ch = Chitin, Cs = Chitosan. (SD = 12%, SE = 4.8% where SD stands for Standard deviation and SE stands for Standard error, respectively); d) Relative quantification of the compounds in the mobile domain based on the J‐ based 2D hCH spectrum. (SD = 13.7%, SE = 6.8%); e) ^1^H‐detected J‐ based 2D hCH spectrum of cell wall; f) Relative intensity of the amino acids in mobile domain (cyan) and rigid domain (brown), SE = 6%; g) Secondary structure chemical‐shift prediction of the amino acids based on the chemical shift deviation (δ). The calculation procedure predicting the secondary structure is given in the material and methods section. The (a) subpart of figure was prepared using biorender.com.

To characterize the *A. fumigatus* cell wall and investigate the influence of CATH‐2 using ssNMR, we divided [^13^C,^15^N] labeled mycelium (See Supporting Information Materials & Methods) into three portions. One portion remained untreated (Apo cell walls) whereas the other two portions were exposed to 5 µM of CATH‐2, either for 1 or 12 h to assess short and long term interactions, respectively (Figure 1a). Each sample was treated with 0.2% para‐formaldehyde (PFA) to halt the growth before cell wall isolation for the ssNMR studies (Figure S4). Previous work^[^ ^31^ ^]^ has shown that such treatment preserves ssNMR spectroscopic signatures seen without treatment.

### Solid‐State NMR Experiments on Apo Cell Walls

To assign the NMR resonances of rigid cell wall components, we combined ^1^H‐detected hCH spectra using dipolar (cross polarization, CP) transfers (Figure 1b) with two‐dimensional (2D) ^13^C‐detected ^13^C‐^13^C correlation experiments (Figure S5). We also acquired ^1^H‐detected 2D CP‐based hCCH data (Figure S6) that revealed additional ^13^C‐^13^C correlations. Experimental parameters for these experiments are given in Tables S1 and S2. Using our peak integration method,^[^ ^11^ ^]^ we quantified both major and minor contributions within the cell wall. Our analysis revealed that α‐1,3‐glucan contributes 37% of the rigid domain, followed by β‐1,3‐glucan (21%) and chitin (17%) (Figure 1c) consistent with previous findings based on ^13^C ssNMR.^[^ ^37^ ^]^ In addition to these major components, we also identified minor contributions of the rigid domain stemming from amino acids (4%), lipids (2%), and chitosan (2%). The presence of chitosan was also confirmed by conducting a ^1^H‐detected ^15^N (hNH) experiment (Figure S7).

To further validate the presence of amino acids in the rigid domain of the cell wall, we conducted ^15^N‐filtered 2D ^13^C‐^13^C correlation experiments^[^ ^38^, ^39^ ^]^ (Figure S8) that revealed protein ^13^C signals at minimal spectral overlap. We observed strong correlations stemming from Valine (V), indicating it is the prominent amino acid in line with previous work.^[^ ^12^ ^]^ We also detected weaker ssNMR signals stemming from Alanine (A), Leucine (L), and Glycine (G) (Figure 1f) which all belong to the most abundant amino acids in hydrophobin proteins^[^ ^40^ ^]^ (Table S3). Interestingly, all amino acids detected by ssNMR in the rigid domain are hydrophobic and tend to adopt α‐helix secondary structures (Figure 1g). These findings raised the question of whether these hydrophobic and α‐helical amino acids are associated with the membrane. However, such spatial proximities were not apparent in ^13^C‐detected 2D CP‐based ^13^C‐^13^C correlation spectra (Figure S9). Instead, we identified intermolecular correlations between valine and chitin as well as between valine and chitosan (VCα‐ Ch8, VCγ–Cs3, and LCβ–Ch8; Figure S9) along with many intermolecular interactions among the polysaccharides, in line with previous results.^[^ ^12^, ^13^, ^37^ ^]^ Taken together, these findings strongly suggest that the amino acids present in the rigid domain are located in close proximity to chitin (Ch) and chitosan (Cs) in hydrophobic domains rather than being integrated into membrane. Such domains may contribute to the stability and robustness of the fungal cell wall.

To assign mobile cell wall species, we acquired ^1^H‐detected scalar hCH spectra revealing through‐bond correlations (Figure 1e) in combination with ^13^C‐detected 2D TOBSY ^13^C‐^13^C correlation spectra^[^ ^41^ ^]^ (Figure S10) and ^1^H‐detected 2D INEPT‐based hCCH spectra^[^ ^33^, ^35^ ^]^ (Figures S11 and S12). Quantification of our ssNMR data^[^ ^11^ ^]^ revealed that polysaccharides contribute 34% of the mobile domain, followed by lipids 35% and amino acids 25% (Figure 1d). Notably and unlike seen in earlier studies,^[^ ^12^ ^]^ all amino acids present in the mobile domain exhibit random‐coil secondary chemical shifts (see, e.g., Ref., [11] Figure 1g). This observation aligns well with predicted structures of hydrophobin proteins (such as RodA to RodG) comprising long unstructured regions (Figure S13).

### The Primary CATH‐2 Targets are the Amino Acids and Polysaccharides in the Mobile Domain

**Figure 2:**
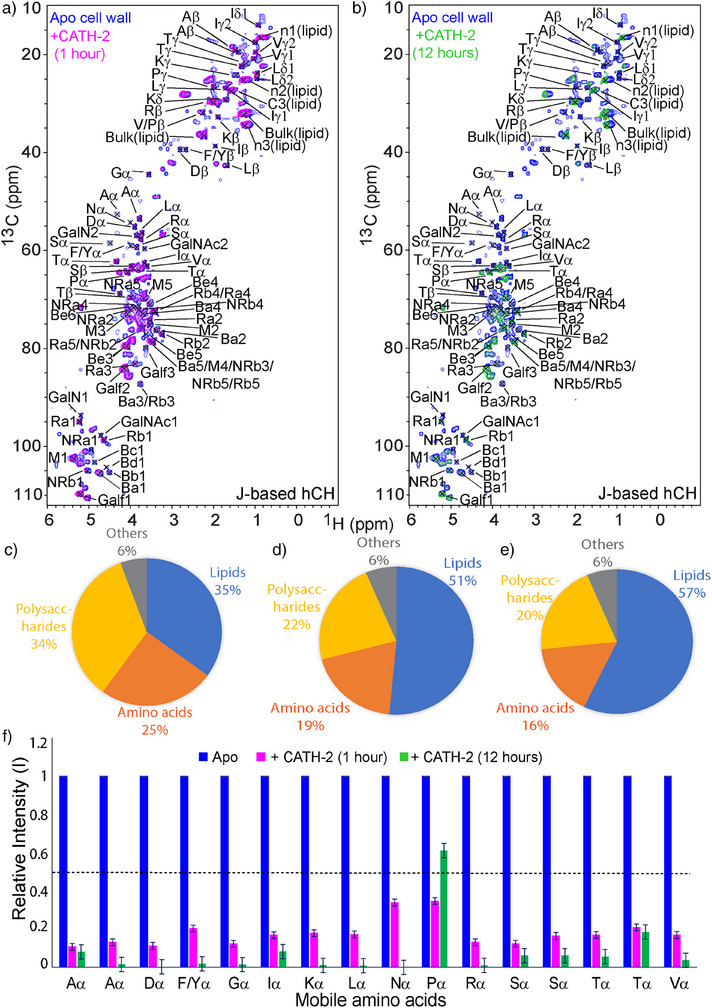
Analysis of mobile cell wall components after CATH‐2 treatment. a) Overlay of ^1^H‐detected J‐ based 2D hCH spectra of Apo (blue) and 1 h treated samples (magenta). b) Spectral comparison of the Apo (blue) and 12 h‐treated (green) sample. See Figures S14 and S16 for additional two and one‐dimensional cut‐outs and slices, respectively. c)–e) Estimation of the molecular composition of the mobile domain based on the peak integration in the J‐ based 2D hCH spectra: c) apo (SD = 13.7%, SE = 6.8%), d) 1 hour of exposure (SD = 18.8%, SE = 9.4%), e) 12 h of exposure (SD = 22.2%, SE = 11%). f) Summary of mobile amino‐acid types and their relative Cα intensities for the three considered samples. Standard errors were determined to 1.8% and 3.5% for 1 and 12 h of the CATH‐2 exposed plots, respectively.

Next, we investigated the mode of action of CATH‐2 (5 µM) to the *A. fumigatus* cell wall using ^1^H‐detected ssNMR. We conducted experiments at both short (1 h) and long (12 h) exposure conditions to observe interactions and possible structural alterations within the cell wall at different incubation times. First, we employed scalar ^1^H‐detected hCH experiments to examine the effects on the mobile domain upon CATH‐2 exposure (Figure 2a,b). Short‐time exposure induced a significant alteration in the hCH spectrum, which exhibited a strong reduction in signals stemming from amino acids and polysaccharides (Figures 2a, S14, S15, S16a, and S16b). This observation suggests that CATH‐2 interacts with mobile components, in particular with amino acids and the polysaccharides within this time frame. Importantly, some specific polysaccharides such as galactosamine (GalN1 and GalN2), N‐acetyl galactosamine (GalNAc1 and GalNAc2), non‐reducing end α‐(1, 3)‐glucan (NRa1, NRa4) and β‐(1, 3)‐glucan (Bc1 and Bd1) completely disappeared from the spectrum (Table S6) which would be compatible with direct or indirect interactions of the peptide with these polysaccharides during this short exposure as already seen for the *S.commune* cell wall.^[^ ^11^ ^]^ Galactosaminogalactan (GAG) is a heteropolysaccharide that is composed of GalN and GalNAc and known to play a crucial role in pathogenesis of the invasive aspergillosis.^[^ ^42^ ^]^ Therefore, these results reveal new insight into the earliest therapeutic targets to treat the pathogenic infections caused by *A. fumigatus*.

Prolonged exposure (12 h) induced stronger changes in the hCH spectrum compared to the 1 h exposure. Specifically, the signals for amino acids dropped to 10% of their original intensity except proline, suggesting a drastic decrease in detectable amino acid content within the mobile part of the cell wall (Figures 2b,f, S15, S16, and S17a). In addition to galactosamine (GalN1 and GalN2) and N‐acetyl galactosamine (GalNAc1 and GalNAc2), certain polysaccharides such as non‐reducing end α‐(1,3)‐glucan (NRa1 and NRa4), reducing end β‐(1,3)‐glucan (Rb3), β‐(1,3)‐glucan (Ba1,Ba2, Ba3, Bb1, Bc1, and Bd1), and Galactofuranose (Galf3) completely disappeared from the spectrum after long exposure (Figure S17b and Table S6). These changes imply that CATH‐2 strongly influences the linkage (C1) and hydroxyl groups of these mobile components within the cell wall over an extended time period.

Additionally, we estimated the relative contributions of mobile components exposed to CATH‐2 (Figure 2c–e). Upon short exposure, the analysis revealed a substantial reduction of polysaccharides and amino acids compared to the apo cell wall. Upon longer term exposure, these effects further increased for both polysaccharides and amino acids. Notably, the relative contribution of the proline signals increased at the longest incubation time, which may indicate a remodeling of the protein network. We conclude that prolonged CATH‐2 exposure leads to even stronger modifications of the relative populations of mobile cell wall components

### The Impact of CATH‐2 Upon the Rigid Cell Wall Domain

**Figure 3:**
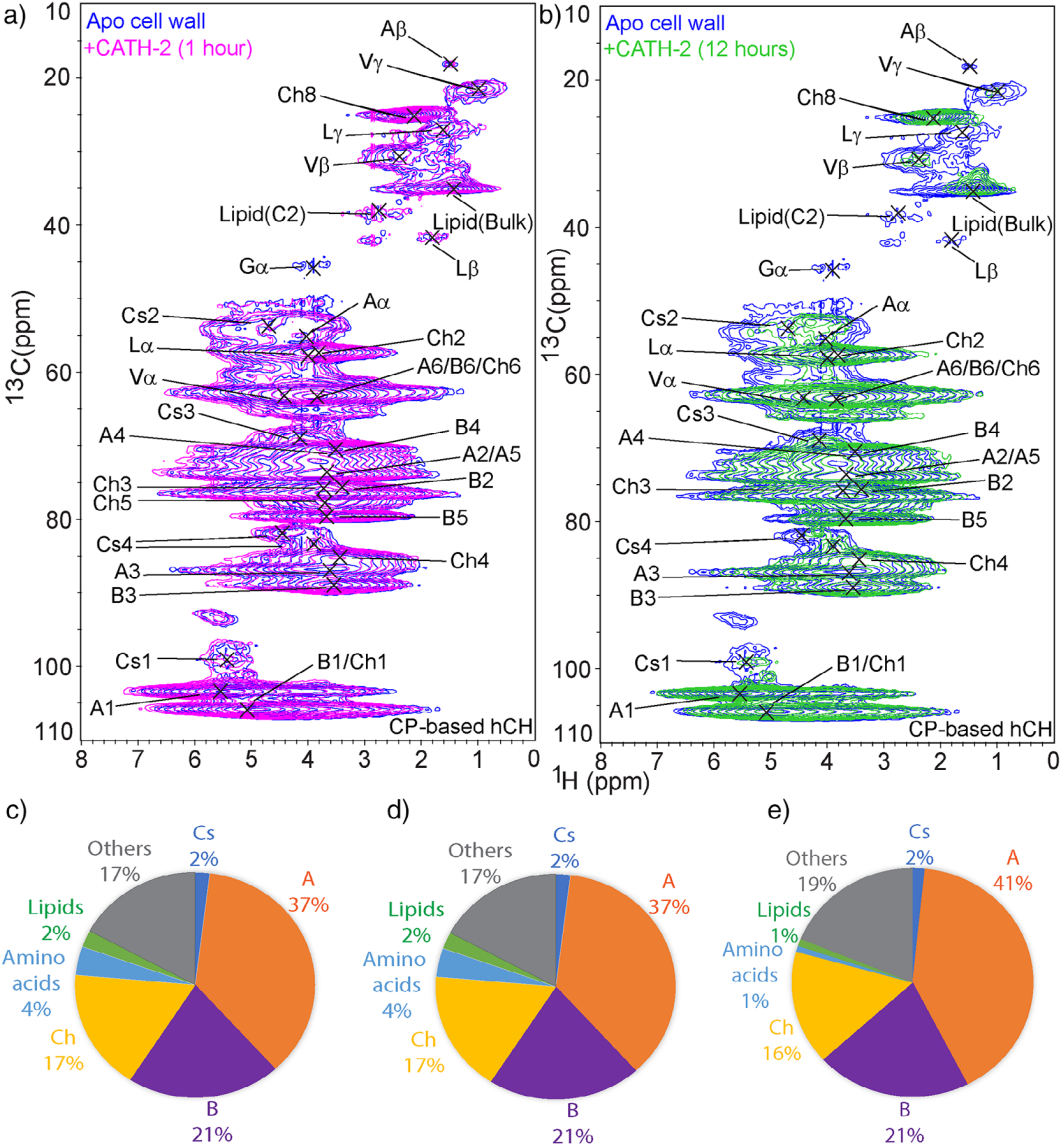
Analysis of rigid cell wall components after CATH‐2 treatment. a) Overlay of ^1^H‐detected CP‐ based hCH spectra of the fungal cell wall. a). Apo (blue) versus 1 hour of exposure (magenta); b) Apo (blue) versus 12 h of exposure (green). See Figure S18 for additional one‐dimaslices. c–e) Estimation of the molecular composition using peak integration in the CP‐ based 2D hCH spectra. c) apo (SD = 12%, SE = 4.8%), d) after 1 h of treatment (SD = 12%, SE = 4.8%), and e) after 12 h of treatment (SD = 15%, SE = 6%); Abbreviations stand for: A = α‐(1,3)‐glucan, B = β‐(1,3)‐glucan, Ch = Chitin, Cs = Chitosan.

After assessing the effects on the mobile domain, we employed ^1^H‐detected CP‐based hCH experiments to examine how CATH‐2 exposure impacts the rigid domain of the cell wall (Figure 3a,b). Short‐time exposure (1 h) did not induce significant spectroscopic changes (Figures 3a and S18) in line with previous findings on *S. commune*.^[^ ^11^ ^]^ In contrast, following a prolonged 12 h exposure to CATH‐2, we observed a substantial reduction in absolute signal intensity of amino acids, lipids, and polysaccharides in the ^1^H‐detected CP‐based hCH spectrum (Figures 3b and S18). On the other hand, the relative contributions of the major rigid cell wall components (α‐1,3‐glucan, β‐1,3‐glucan, and chitin) did not alter for both short‐ and long‐term CATH‐2 exposure compared to the untreated case (Figure 3c–e). These findings suggest that the structural organization of the rigid polysaccharides is only mildly impacted by CATH‐2 at short exposure and remains still largely intact at longer times. These findings led us to hypothesize that CATH‐2 perturbs the cell wall at longer exposure by direct or indirect interactions as well as possibly by intracellular processes including altered biosynthesis after membrane permeabilization which has been observed in *C. albins*.^[^ ^21^ ^]^

### CATH‐2 Induces a Larger Water Interface in the Rigid Domain of the Cell Wall

**Figure 4:**
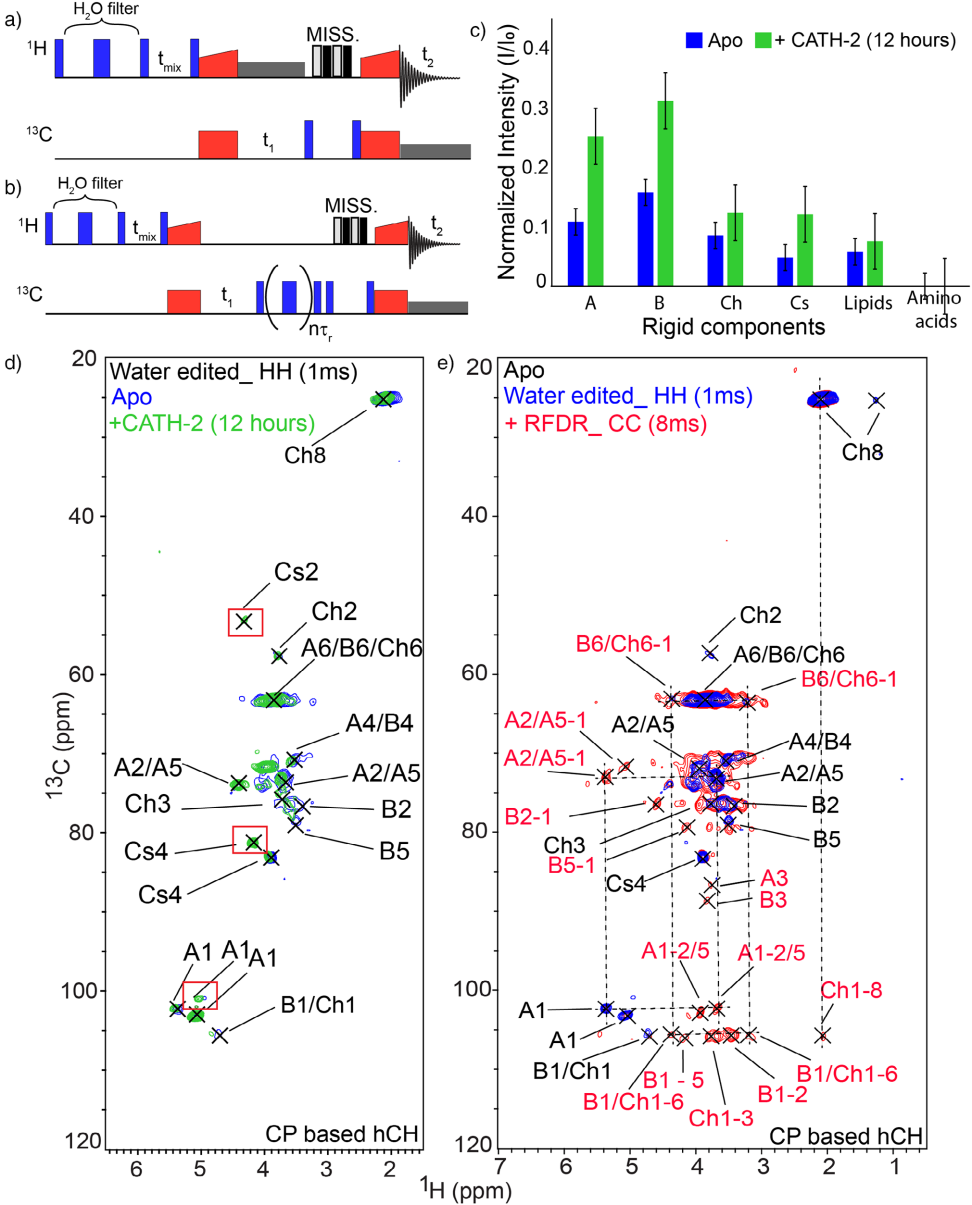
Water interface of *A. fumigatus* cell walls as seen by ssNMR: a) Pulse sequences for water‐edited hCH experiments; b) Water‐edited hCH with RFDR^[^ ^17^ ^] 13^C‐^13^C mixing experiments. c) Intensity ratios of the water edited spectra normalized to the regular spectrum of the rigid polysaccharides. Standard errors are 2.2% and 4.7% for apo and 12 h of the CATH‐2 exposed data, respectively. d) Overlay of water edited 2D hCH spectrum obtained on Apo (Blue) and CATH‐2 (12 h, Green) treated samples using 1 ms of ^1^H‐^1^H mixing. Additional correlations that only appeared in the CATH‐2 treated sample are indicated by red boxes. e) Comparison of water‐edited 2D hCH spectra with 1 ms ^1^H–^1^H mixing times using 0 ms (blue) and 8 ms (red) ^13^C–^13^C mixing. Additional ^13^C–^13^C correlations are indicated by dashed lines.

Next, we investigated whether extended exposure to CATH‐2 might also influence the water accessibility of the cell wall. Previously, ^13^C‐detected H_2_O edited ssNMR experiments^[^ ^43^ ^]^ have been used to probe and analyze H_2_O‐surfaces of proteins in lipid bilayers. To increase the spectroscopic sensitivity and resolution, we implemented ^1^H‐detected variants of the original pulse schemes.^[^ ^43^ ^]^ We note that earlier reports proposed related ssNMR experiments to probe membrane‐proteins using selective pulses^[^ ^44^ ^]^ or for the selective detection of Intermediate‐Amplitude motion.^[^ ^45^ ^]^ Using this pulse scheme (Figure 4a), we compared the water accessibility of the cell wall components after long‐term CATH‐2 exposure with that of an untreated (apo) cell wall.

First, we measured the water‐edited build‐up curves for cell walls before and after peptide treatment by varying the length of the ^1^H–^1^H mixing time, allowing to follow polarization transfer from H_2_O molecules to polysaccharides. Both cell wall samples showed sigmoidal build‐up curves with faster and more efficient transfer rates for the long‐term exposed cell wall sample (Figure S19). Comparison of the water‐edited 1D hCH spectrum at maximum transfer time with the regular 1D hCH spectrum of the untreated (apo) cell wall, suggest that approximately 13% (SD = 5.4%, SE = 1.2%) of the total rigid cell wall domain is accessible to water (Figure S20a). In contrast, prolonged CATH‐2 exposure cell wall leads to an increase to approximately 30% (SD = 11%, SE = 2.5%) of the rigid cell wall domain becomes water‐accessible (Figure S20b).

Furthermore, to investigate the hydration level at atomic level, we measured a 2D H_2_O‐edited hCH with 60 ms of ^1^H–^1^H mixing time on both the samples (Figure S20c,d). The I/I_0_ intensity ratios between the water‐edited 2D hCH with 60 ms mixing (I) and regular 2D hCH (I_0_) spectra served as relative measure of the water retention levels of each rigid component. The high‐intensity ratio (I/I_0_) of 0.16 of β‐glucan (B) revealed that it is more hydrated compared to α‐glucan (A) (0.11), chitin (Ch) (0.09) and chitosan (Cs) (0.05) in the untreated cell wall (apo) (Figure 4c), which is consistent with the previous findings.^[^ ^12^, ^13^, ^37^ ^]^ In contrast, the I/I_0_ ratios of β‐glucan (B), α‐glucan (A), chitin (Ch) and chitosan (Cs) increased to 0.31, 0.25, 0.12, and 0.12, respectively, upon extended CATH‐2 treatment (Figure 4c). These higher ratios (I/I_0_) suggest that each of these components has become significantly more accessible to water. Interestingly, neither of the samples showed any amino acid signals in the water‐edited spectrum, implicating that the amino acids are likely buried within hydrophobic polysaccharide pockets that are entirely inaccessible to water. These findings lead us to hypothesize that CATH‐2 may disrupt the intermolecular interactions of the polysaccharides and thereby alters the integrity of the rigid domain of the cell wall.

Previous studies have reported chitin, along with α‐glucan and β‐glucan, as major components of the rigid cell wall, characterizing a high degree of the polymorphism within their crystalline structures.^[^ ^46^, ^47^ ^]^ Correspondingly, the ^1^H line width of the polysaccharides found in the rigid domain of the *A. fumigatus* are extremely broad (Figure 3a,b), such as the α‐glucan (A) at 4.74 ppm on 700 MHz, even when measured at a fast spinning rate of 60 kHz. To examine the proton chemical shifts of the polysaccharide species that are in close proximity to water, we measured the water‐edited version of the ^1^H‐detected 2D hCH experiment with a short (1 ms) ^1^H–^1^H mixing time before and after CATH‐2 exposure (Figures 4d and S21). This analysis revealed remarkably narrow proton chemical shifts of the polysaccharides such as two monomorphic, possibly crystalline‐like, forms of α‐glucan(A) that are characterized by a ^1^H line width of 0.19 and 0.15 ppm. We also observed additional correlations from chitosan (Cs) and α‐glucan (A) after extended CATH‐2 exposure (Figure 4d, green, indicated by red boxes) compared to the untreated cell wall sample. These results again confirm that CATH‐2 increases the water accessibility of the polysaccharides. To further refine our analysis, we modified the pulse sequence by adding a ^13^C^13^C‐RFDR mixing^[^ ^17^ ^]^ block just before the detection phase (Figure 4b). This modification enabled the intramolecular assignment of the monomorphic / crystalline polysaccharides, providing a more comprehensive understanding of their structure (Figure 4d). In Table S8, assignments on the basis of these intramolecular correlations are summarized. Comparison to previous ssNMR studies on chitin,^[^ ^48^ ^]^ suggested a prominent occurrence of the β‐chitin isoform in the water‐exposed cell wall interface.

## Discussion

**Figure 5:**
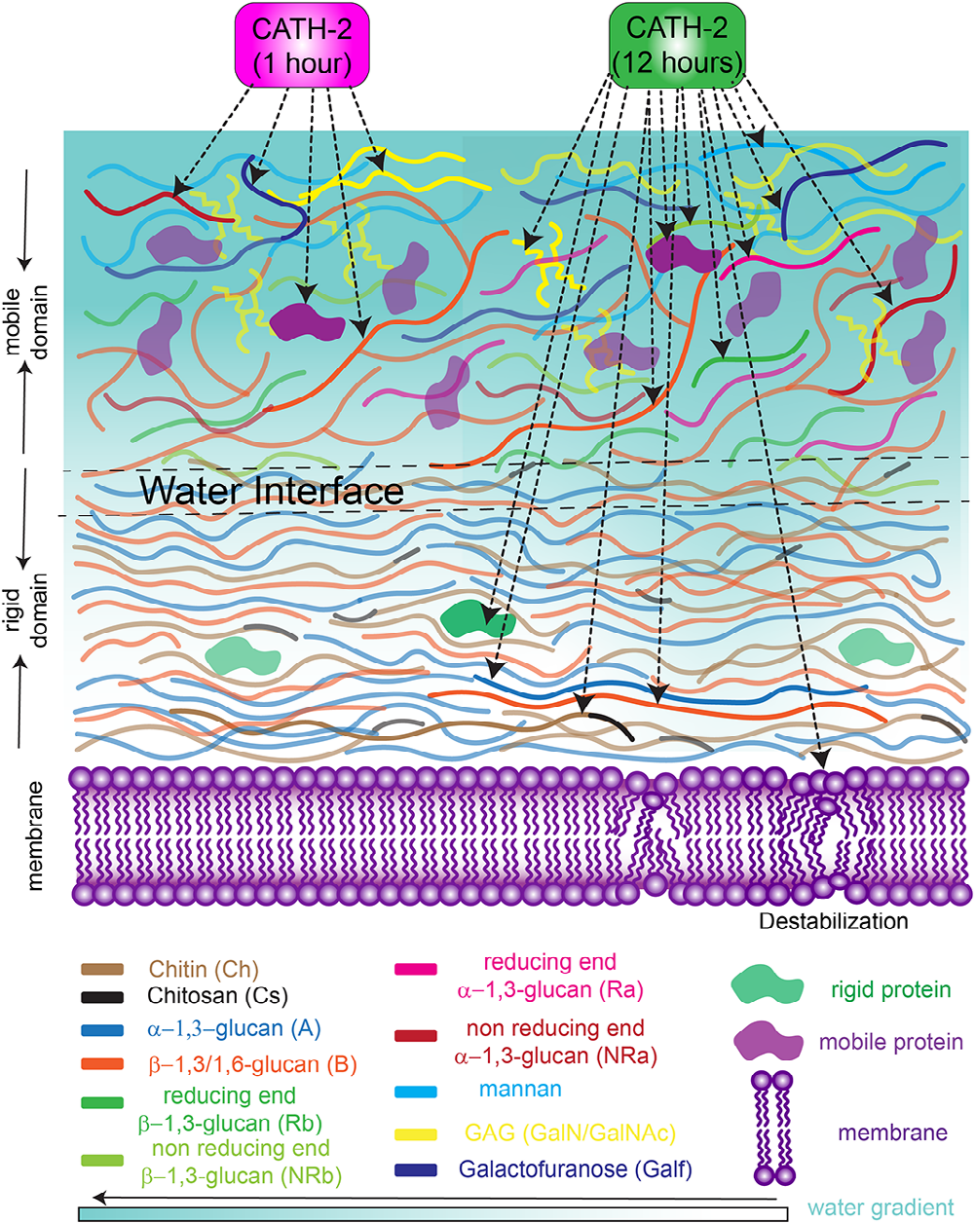
Schematic representation of the *A. fumigatus* cell wall showing step‐wise targeting by CATH‐2 upon short‐term (magenta) and long‐term exposure (green).

We have highlighted the capability of ^1^H‐detected ssNMR to advance our understanding into the organization of the *A. fumigatus* cell wall and its interaction with the antimicrobial peptide CATH‐2 (see Figures S23–S26 for a summary of polysaccharides and a schematic of lipids investigated here). Our experiments confirmed that the amino acids within the rigid domain adopt an α‐helical secondary structure and are located in close proximity to chitin and chitosan. In contrast, the amino acids within the mobile domain are unstructured. Possibly these are part of the unstructured regions of hydrophobins. In addition, we were able to shed light into the atom‐specific interaction sites for CATH‐2 in the cell wall of *A. fumigatus* during short and long term exposure (Figure 5). The spectral changes observed for amino acids and some specific polysaccharides present within the mobile domain during short exposure suggest that CATH‐2 already penetrates into the mobile domain after a short exposure time. During longer exposure, CATH‐2 not only impacts the mobile domain components that were initially observed during the short exposure, but also influences rigid domain components along with the bulk lipid. This confirms that CATH‐2 is able to also impact large parts of the hydrophobic rigid cell wall as well as the membrane by interacting either directly or indirectly with both the mobile and rigid domains during prolonged exposure. Further work, for example by using labeled CATH‐2 peptides may elucidate to which extent direct effects (such as peptide binding) and indirect effects (e.g., alterations in biosynthesis after membrane permealization^[^ ^21^ ^]^) contribute to the observed cell wall changes. For such studies, time‐resolved cell wall studies that track biosynthesis during peptide exposure would be useful and could be combined with high‐sensitivity ssNMR methods to study isotope‐labeled CATH‐2 peptides in situ.

Our data are compatible with a reorganization of the rigid cell wall that leads to enhanced penetration of H_2_O. Such H_2_O “channels” may be needed to reach and modulate the membrane layer. Furthermore, we observed an increase in water accessibility in the rigid components after prolonged exposure, which suggests that CATH‐2 may disrupt the covalent linkages between the polysaccharides. Close to this water interface, polymorphic polysaccharides seem to adapt monomorphic, possibly crystalline structures. This notion offers a potential mechanism of action of CATH‐2 that targets specific cell wall polysaccharides and proteins, with membrane permeabilization to inhibit the growth of *A. fumigatus*.

## Conclusion

In summary, this study highlights the potential of ssNMR for investigating cell wall interactions with the antifungal compound CATH‐2 at atomic level in situ (i.e., *A. fumigatus* mycelium instead of the isolated cell wall). Our data shows time‐dependent interactions between CATH‐2 and the cell wall which could promote the ability of the peptide to reach the plasma membrane. This might be especially relevant at lower peptide concentrations where CATH‐2 might be trapped in the cell wall or delayed in its transfer to reach the membrane. Alternatively, or in parallel, the interactions between CATH‐2 and cell wall components might be part of the antimicrobial mechanism, e.g., by destabilizing the cell wall via reorganization of its structure and increasing its water content. In a broader sense, our current findings may pave the way for future research to fully unravel the role of fungal cell wall components in protection and killing by antimicrobial peptides.

## Conflict of Interests

The authors declare no conflict of interest.

## Supporting information



Supporting Information

## Data Availability

The data that support the findings of this study are available from the corresponding author upon reasonable request.
