# Supplementary material for: Solid‐State NMR Reveals Reorganization of the Aspergillus fumigatus Cell Wall Due to a Host‐Defence Peptide
Source: Angew Chem Int Ed Engl. 2025 Jul 16;64(35):e202509012. doi: 10.1002/anie.202509012 (PMC12377432; doi:10.1002/anie.202509012)
Supplement: Supplementary file 1 — Supporting Information [file ANIE-64-e202509012-s001.pdf]

Supplementary Information

Solid-state NMR Reveals Reorganization of the *Aspergillus fumigatus* Cell Wall Due to a Host-Defence Peptide

Supporting Figures 1 – 26

Supporting tables 1 – 12

Material and Methods

Supporting references

Supporting Figures

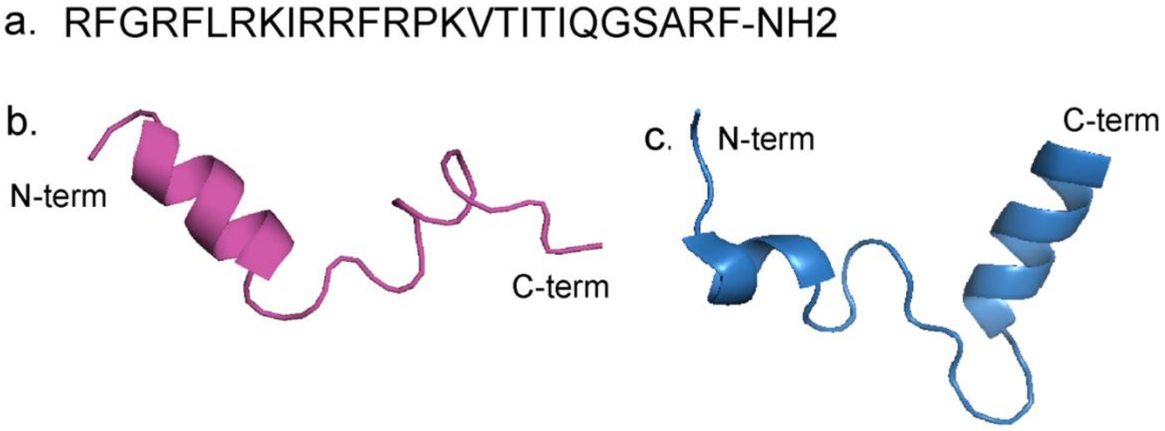

Fig. S1. (a). Amino acid sequence of CATH-2. **(b).** Structure of CATH-2 from Alphafold 2.0[1]. **(c).** Solution state NMR structure of CATH-2 in TFE (PDB:2GDL)[2].

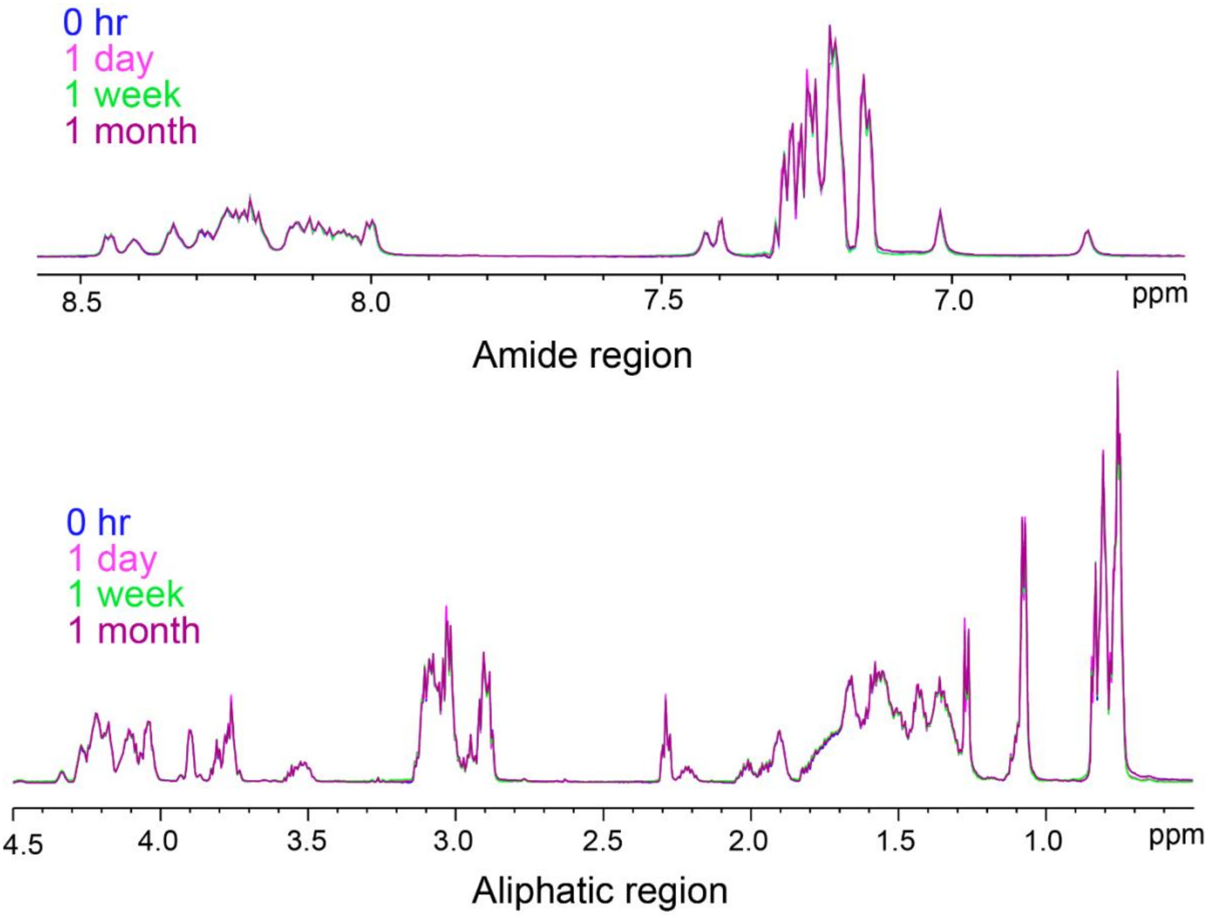

Fig. S2. Stability of CATH-2 at 37⁰C for 1 month as seen from ^{1}H NMR solution-state NMR. Amide (top) and aliphatic (bottom) ^{1}H resonances remain unaltered after incubating at 37⁰C for up to one month.

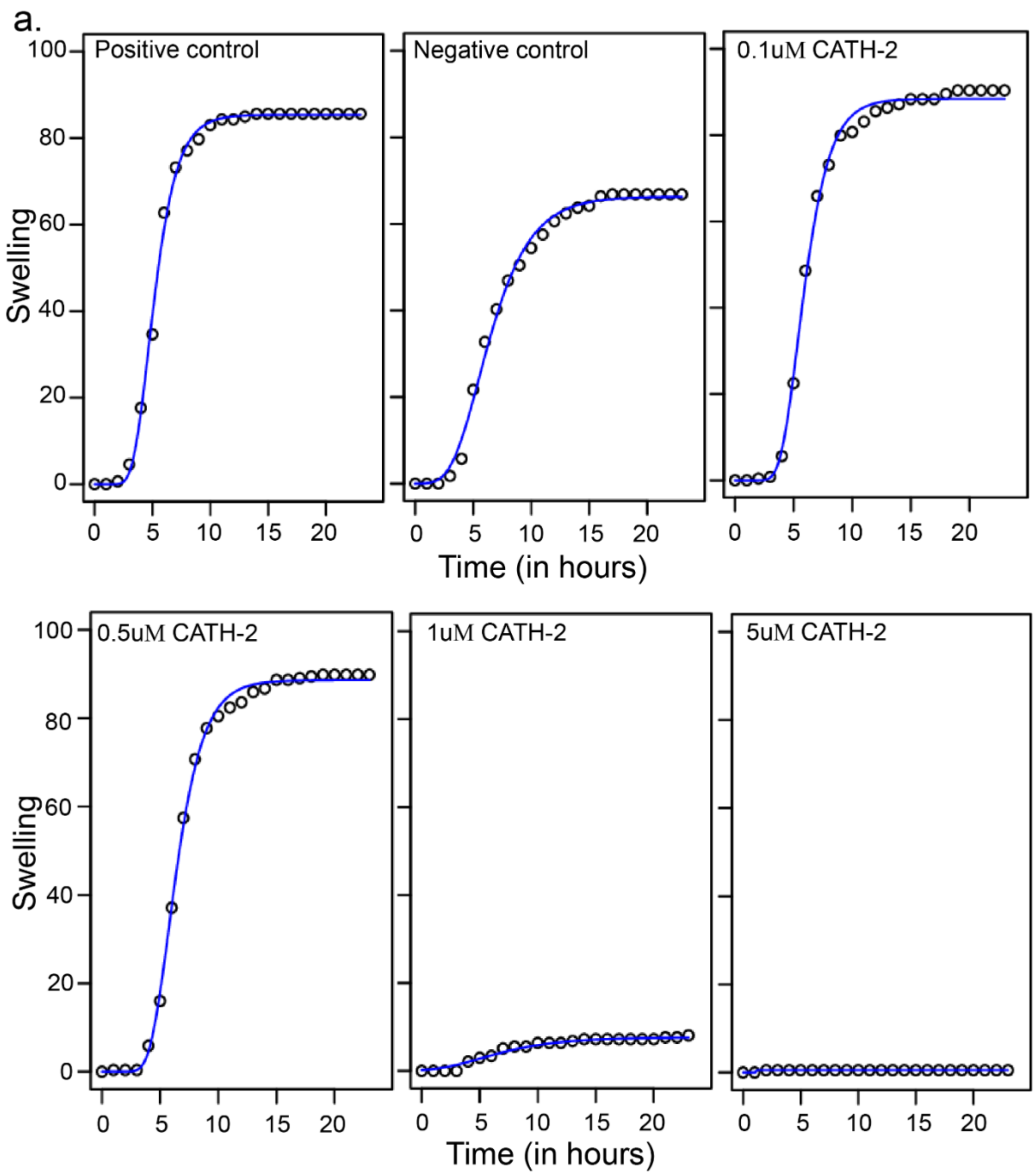

a.
Positive control
Negative control
0.1uM CATH-2
0.5uM CATH-2
1uM CATH-2
5uM CATH-2
Swelling
Time (in hours)
0
20
40
60
80
100
0
5
10
15
20

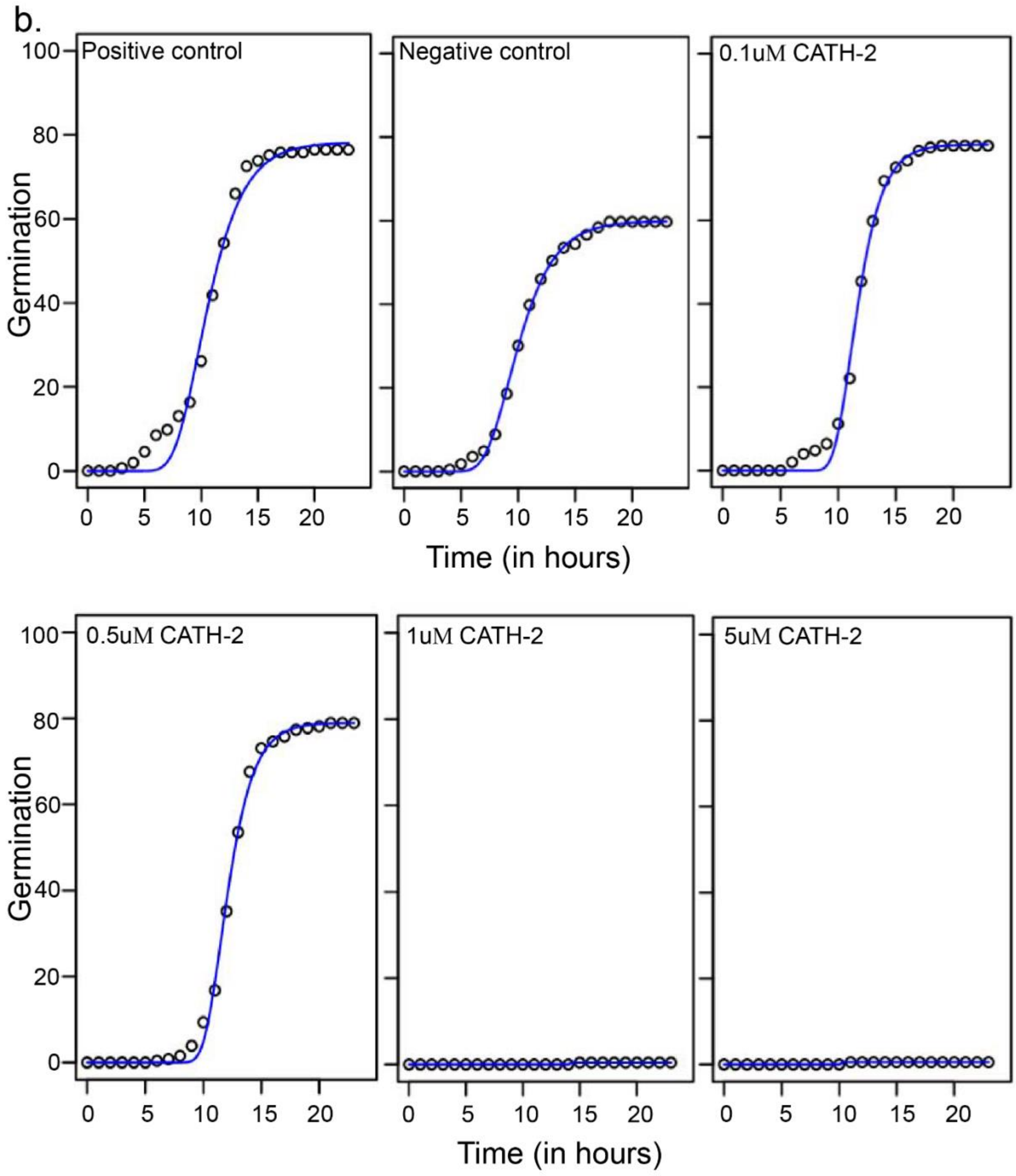

Fig. S3. Effect of CATH-2 on swelling and germ tube formation of *A. fumigatus*. (a). Growth curve for the different conditions showing the percentage of the followed spores that are swelling overtime. **(b).** Growth curve for the different conditions showing the percentage of the followed spores that are forming germ tube overtime.

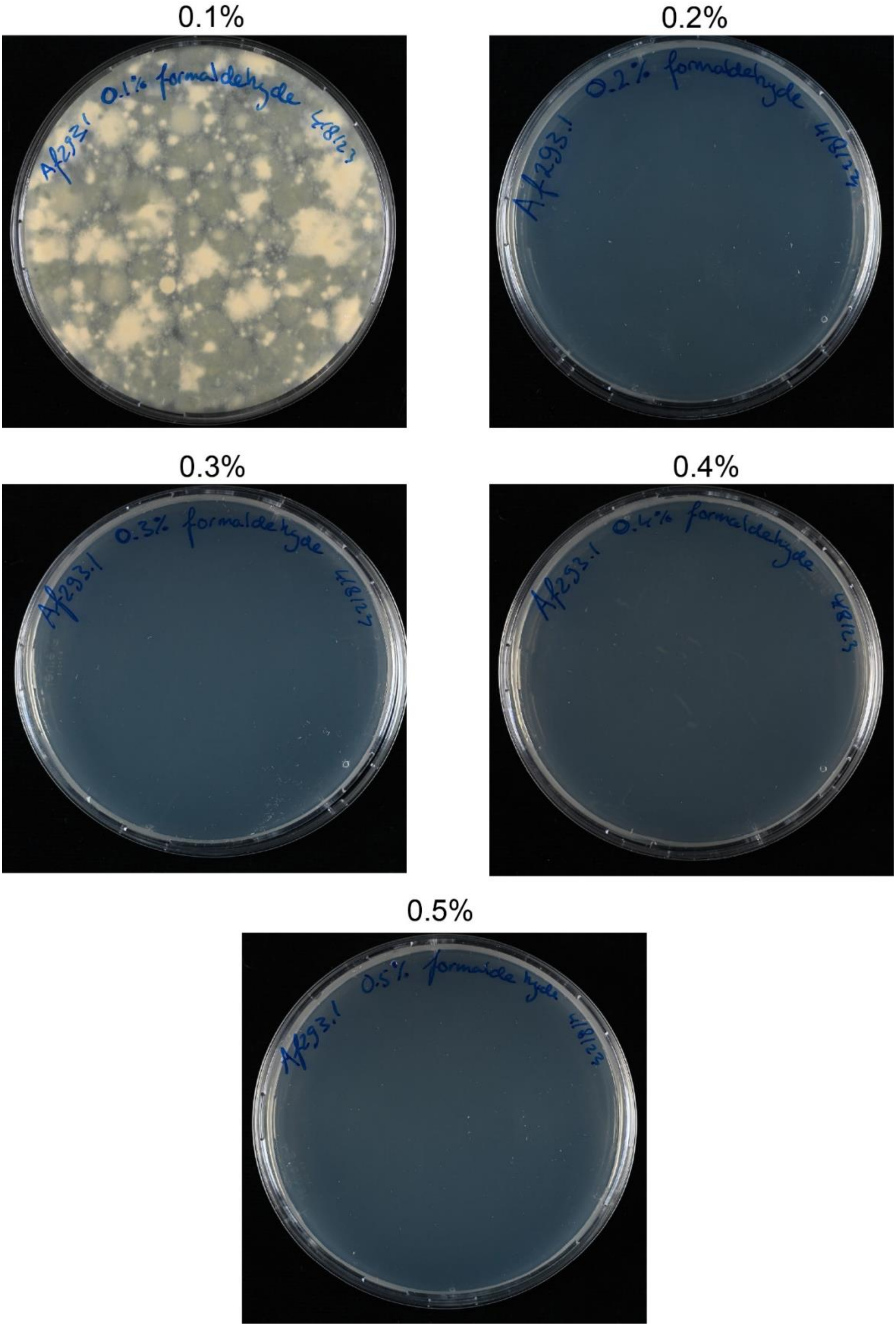

Fig. S4. Para-formaldehyde (PFA – 0.2%) inactivates *A. fumigatus*. *A. fumigatus* spores were plated on PDA media after 1 h treatment with 0.1-0.5% of PFA respectively.

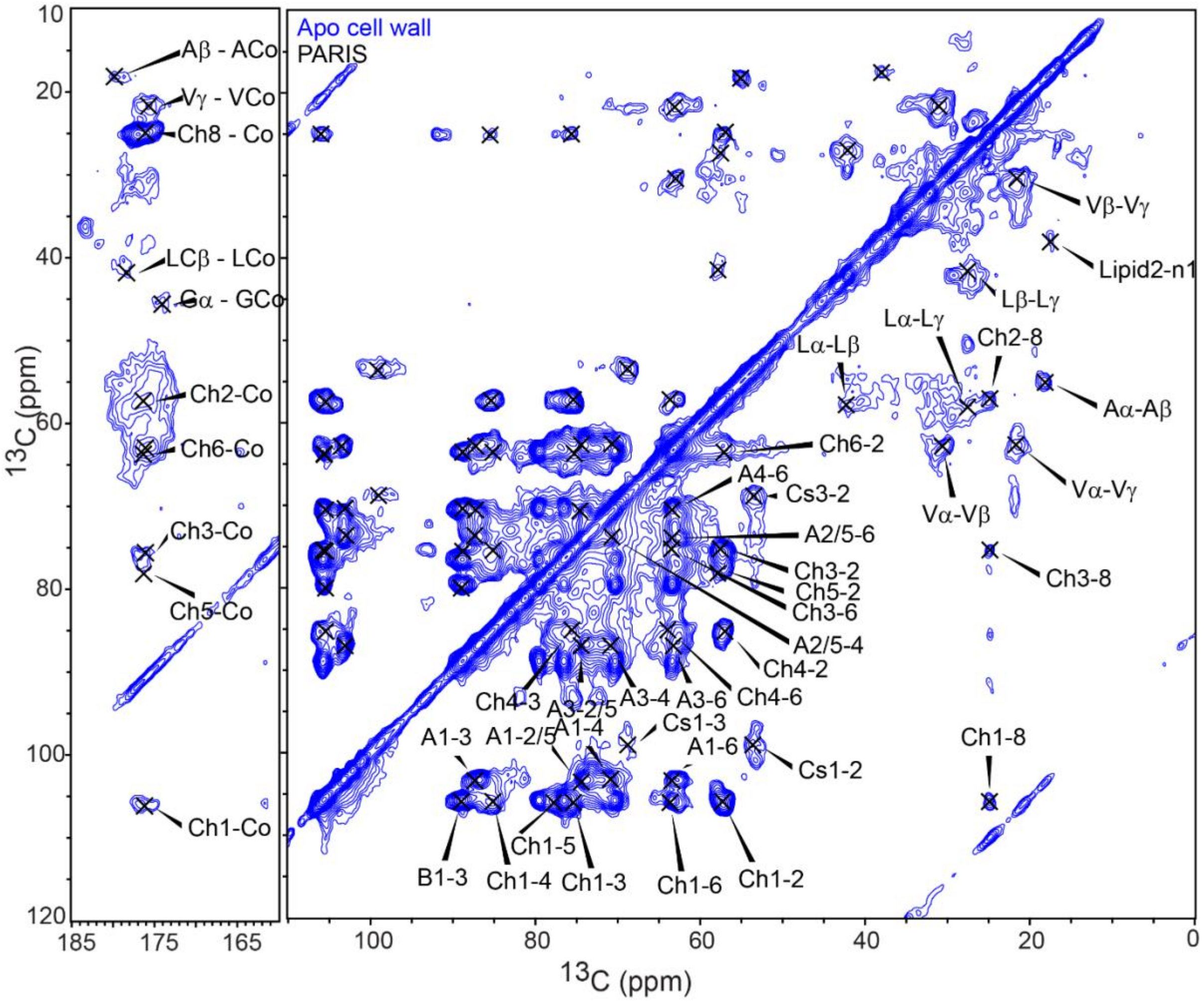

Fig. S5. ^{13}C based 2D CC PARIS experiment[3] showing through-space ^{13}C correlations including intra-amino-acid cross peaks. The mixing time was set to 30 ms.

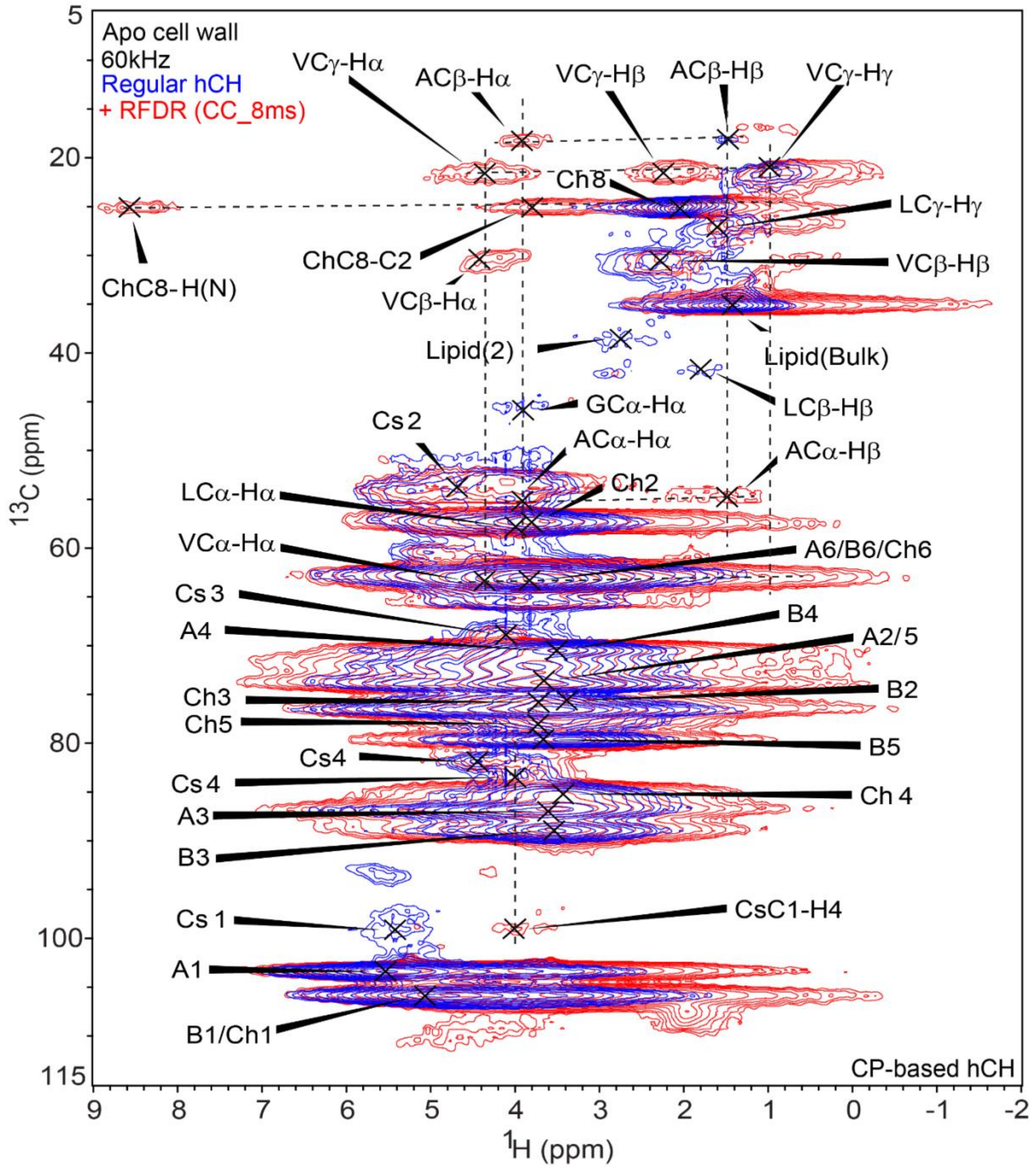

Fig. S6. Overlay of ^{1}H-detected 2D hCH and hCCH spectra using 8 ms RFDR[4] mixing.

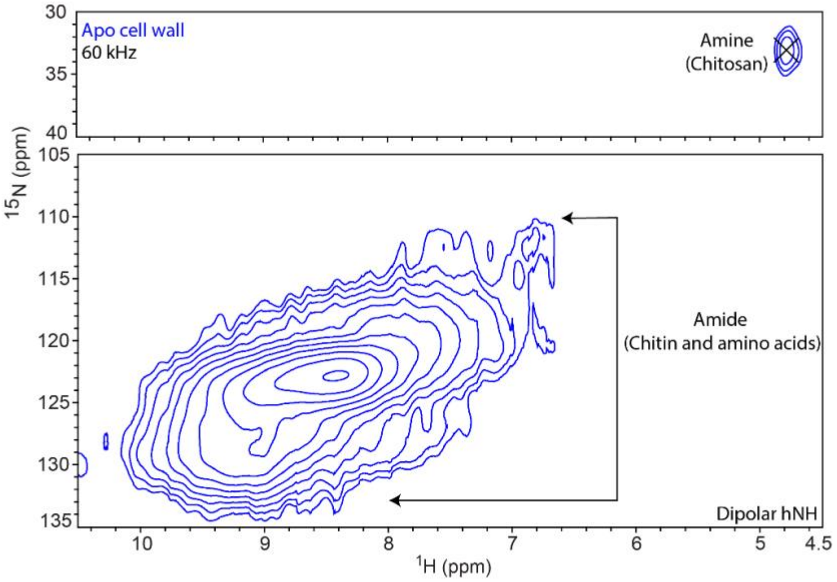

Fig. S7. ^{1}H-detected CP based hNH[5].

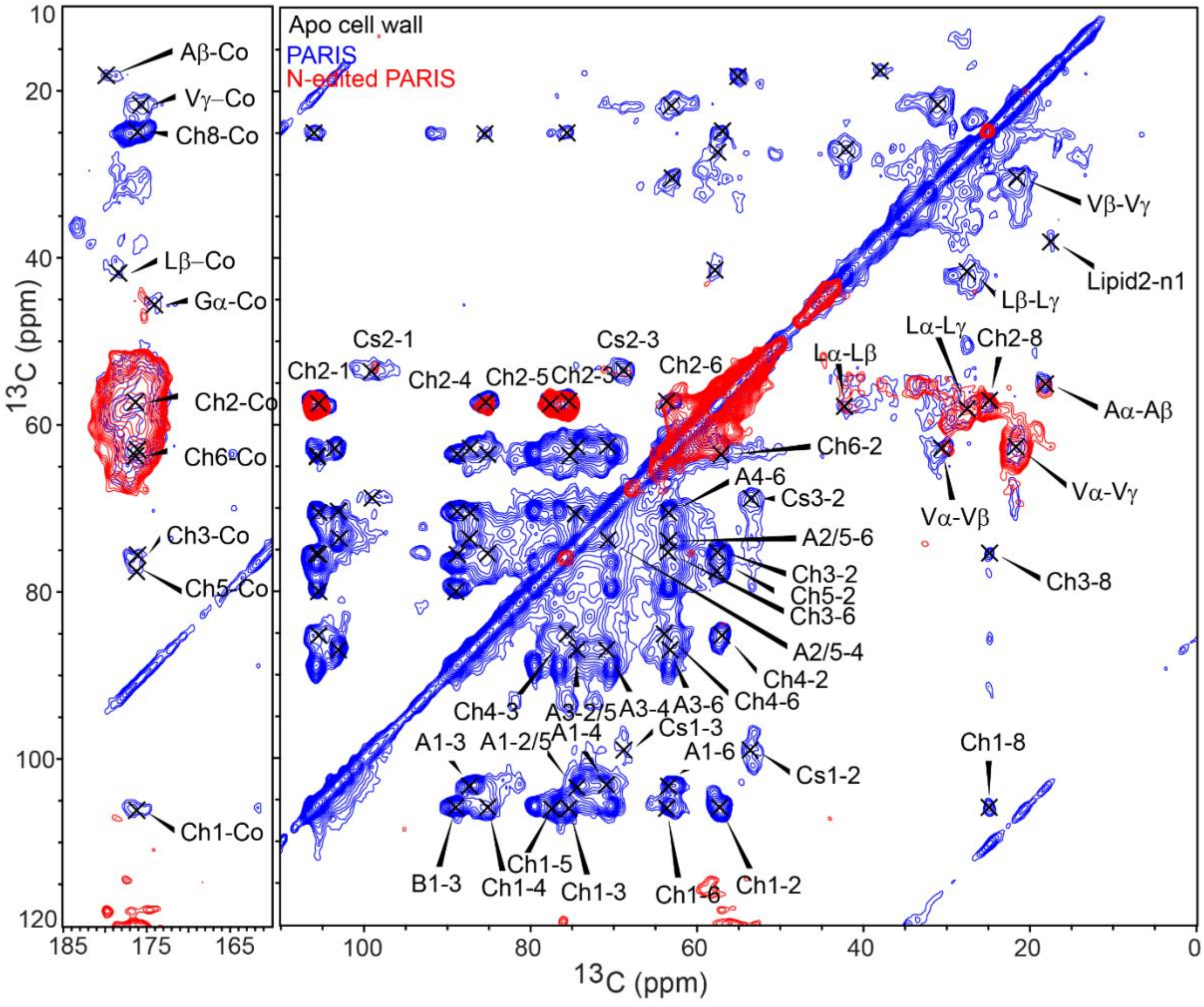

Fig.S8. Overlay of 2D CC PARIS (blue) and ^{15}N-edited 2D CC PARIS (red) experiments. The ^{15}N-edited experiment filters out correlations between chitin, chitosan and amino acids.

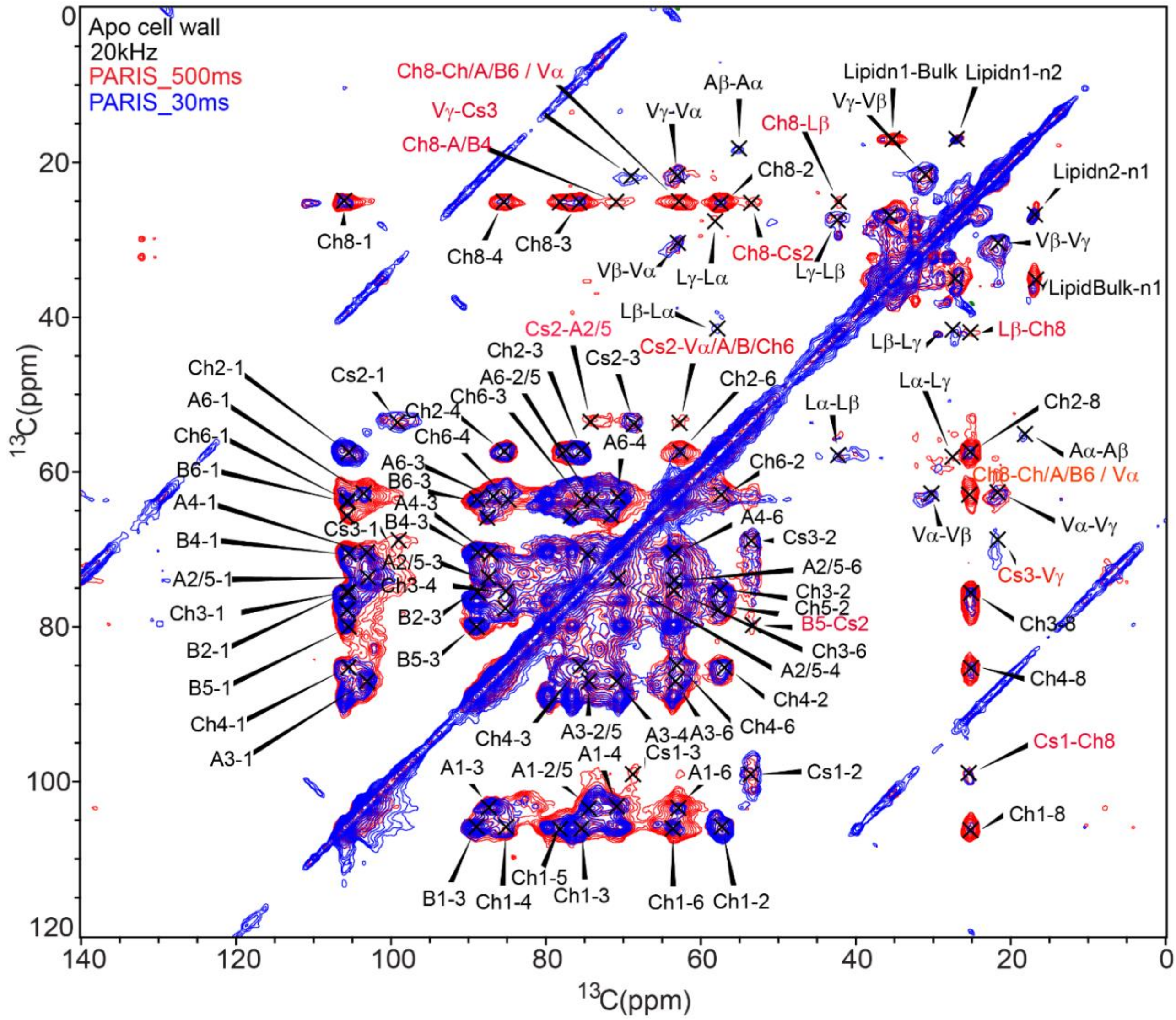

Fig. S9. Overlay of ^{13}C based 2D CC PARIS[3] using different mixing times (50 ms, blue; 500 ms, red). At long mixing times, inter-molecular correlations appear.

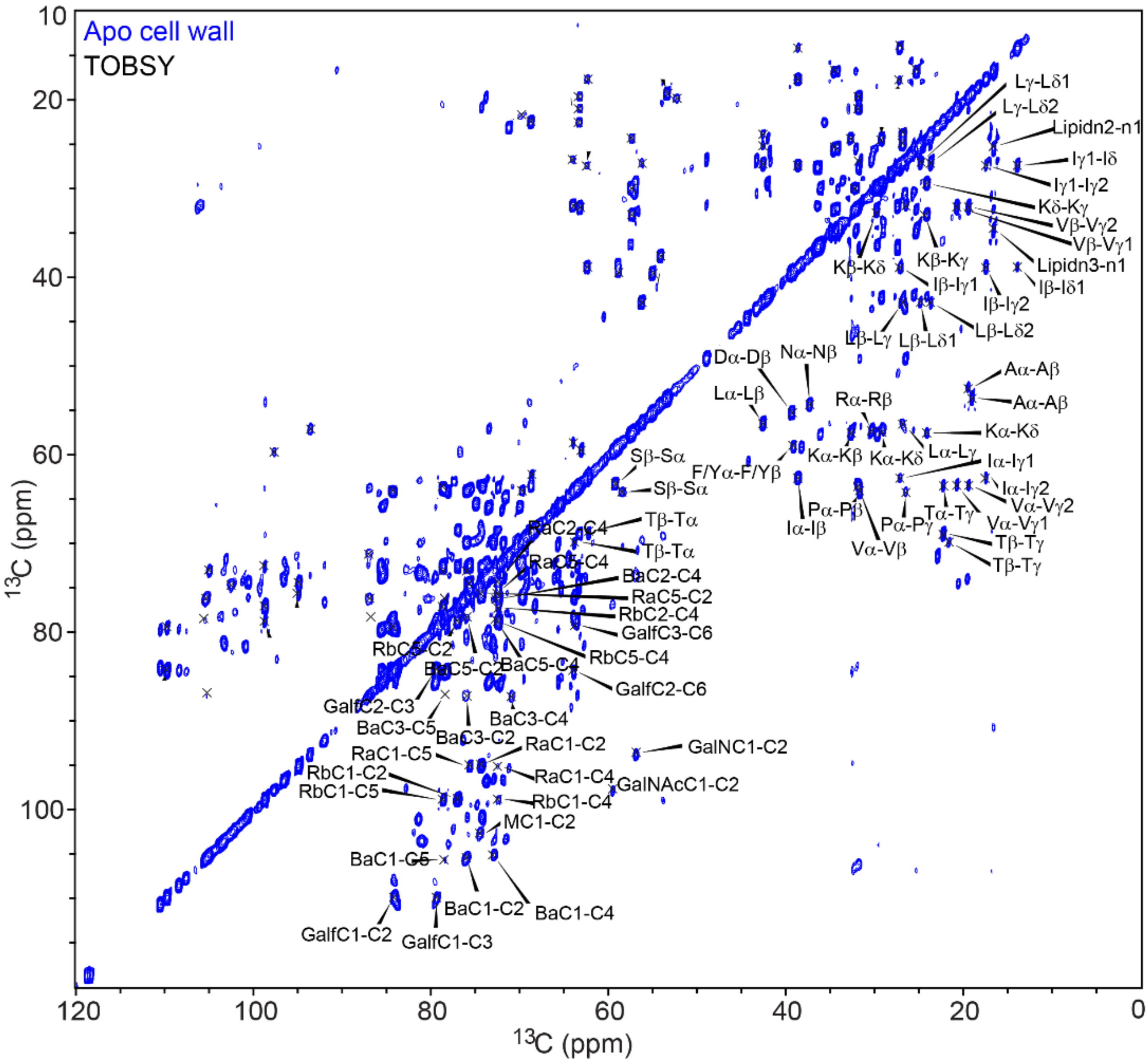

Fig. S10. ^{13}C -based 2D CC TOBSY[6] showing through-bond correlations of the mobile components.

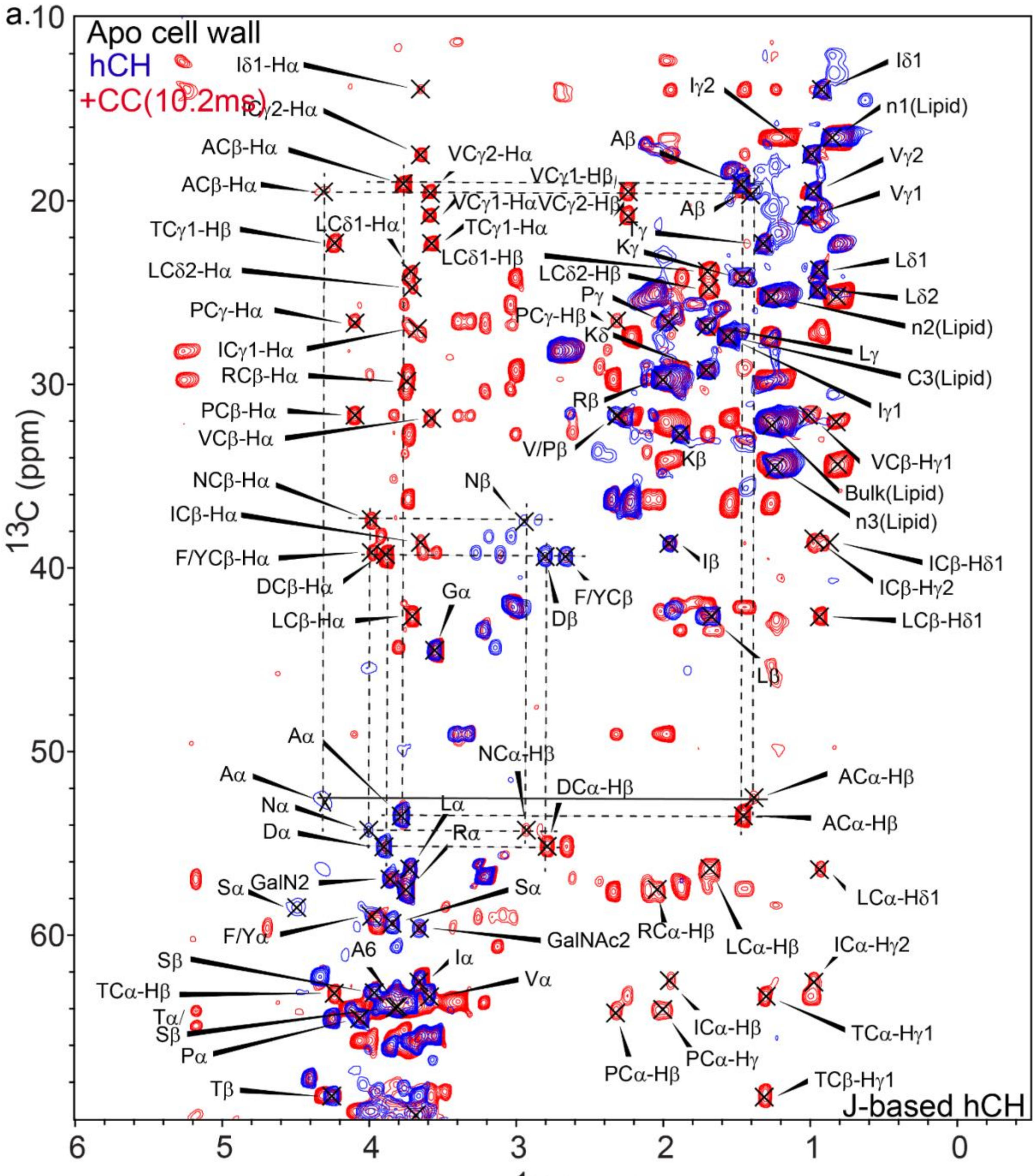

Fig. S11. Overlay of ^{1}H -detected 2D INEPT (blue) and ^{1}H-detected 2D INEPT based hCCH (red, 10.2 ms WALTZ mixing)[7] in the aliphatic region.

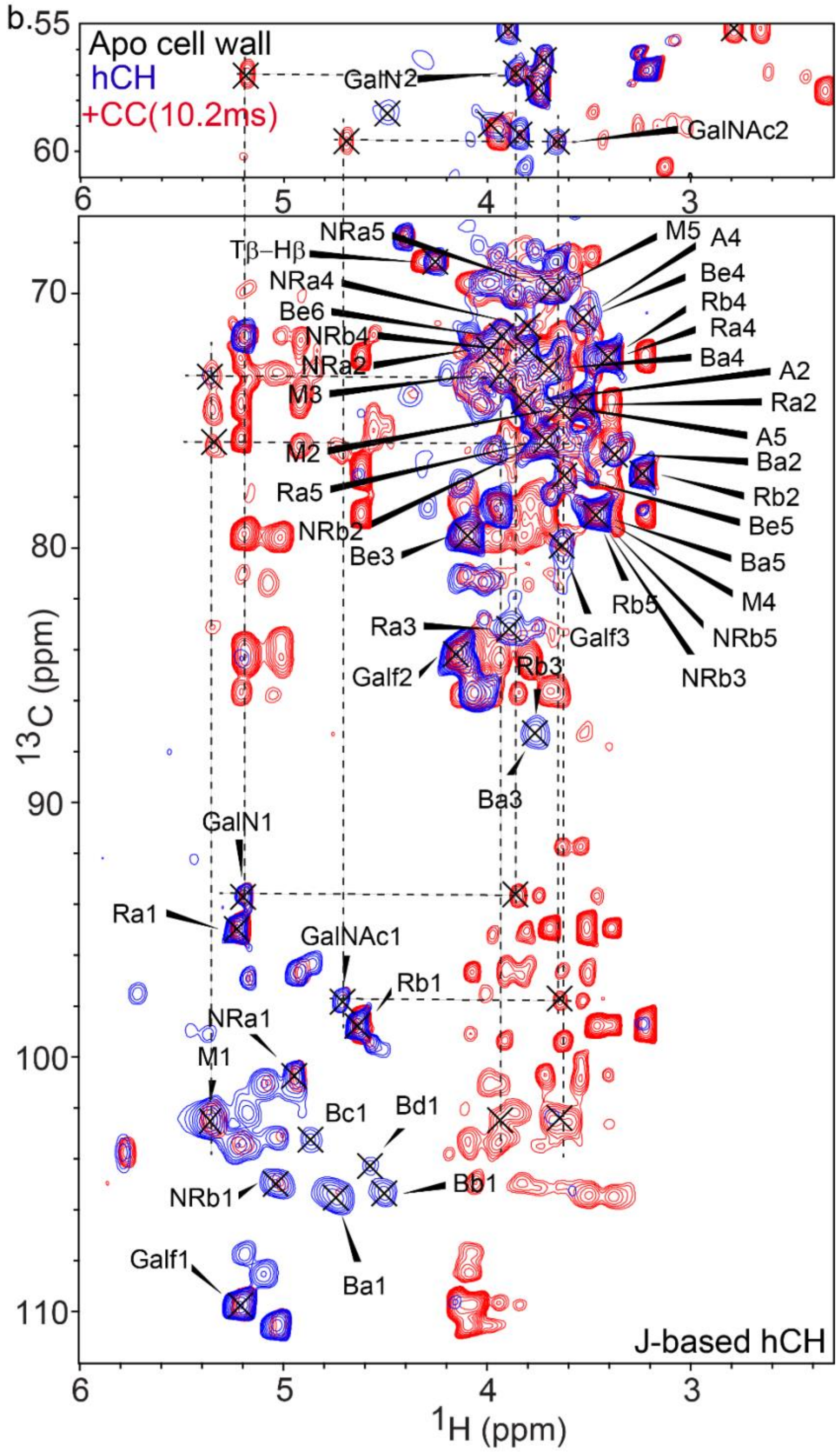

Fig. S12. Overlay of ^{1}H -detected 2D INEPT (blue) and ^{1}H-detected 2D INEPT based hCCH (red, 10.2 ms WALTZ mixing)[7] in the sugar region.

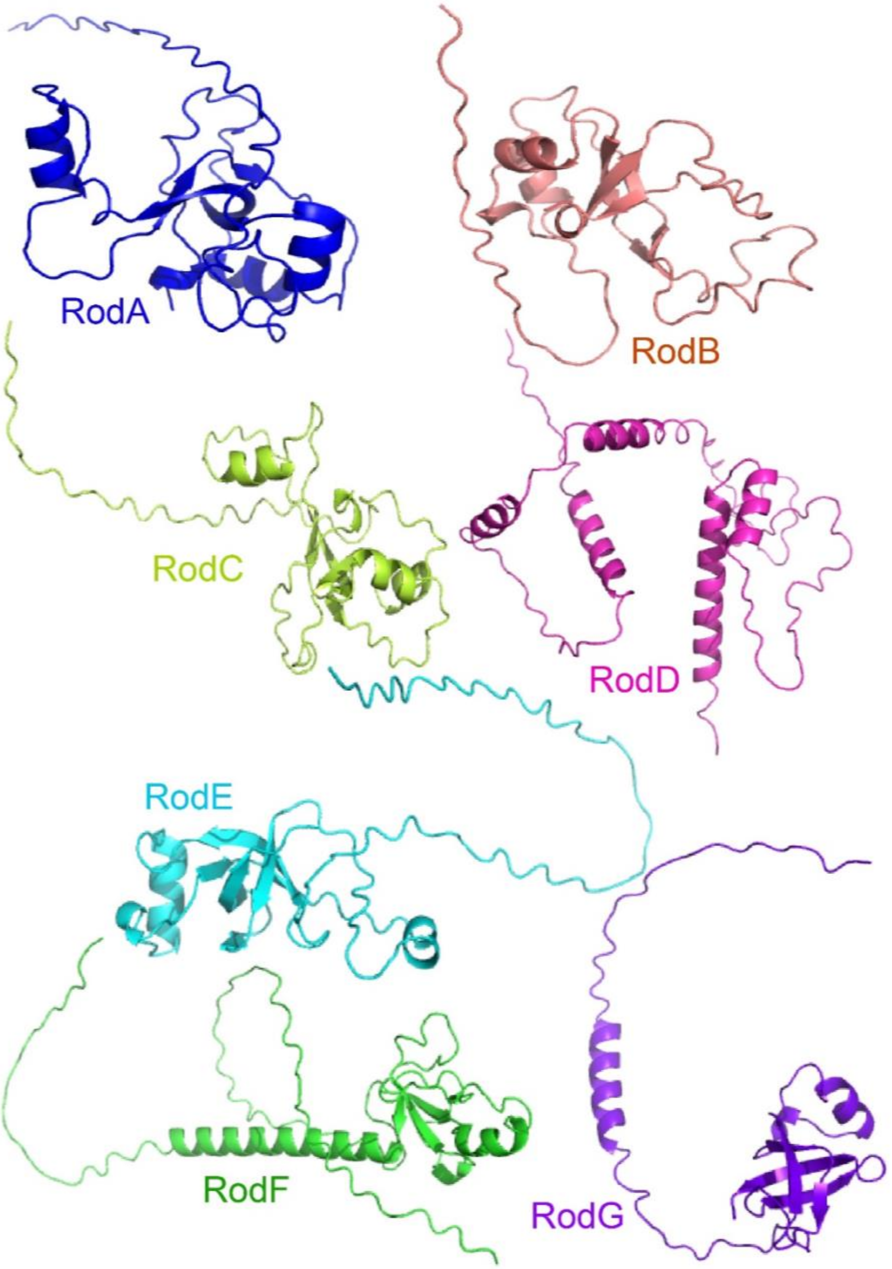

Fig. S13. Hydrophobin structures from Alphafold 2.0[1].

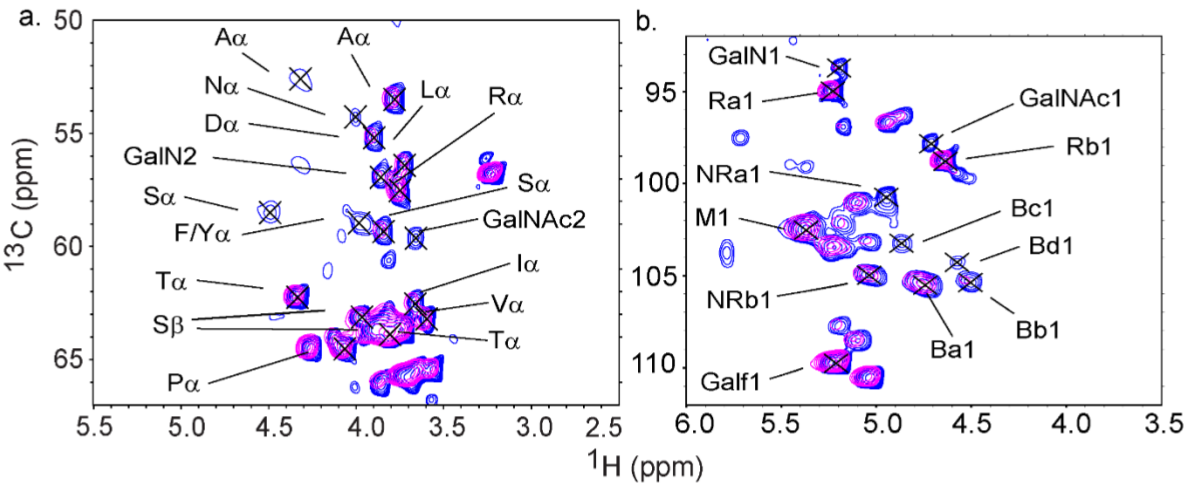

Fig. S14. (a) Spectral Cut-outs taken from **(Fig 2a)** displaying characteristic amino acid (a) and polysaccharide (b) correlations. Apo sample: blue. CATH-2 (1 hour) treated sample: magenta.

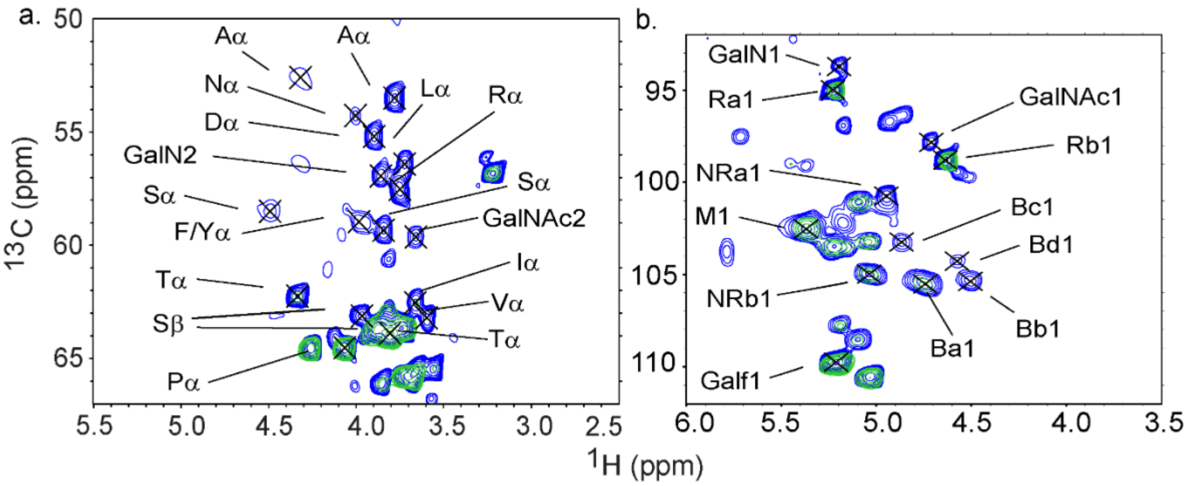

Fig. S15. (a) Spectral Cut-outs taken from **(Fig 2b)** displaying characteristic amino acid (a) and polysaccharide (b) correlations. Apo sample: blue. CATH-2 (12 hour) treated sample: green.

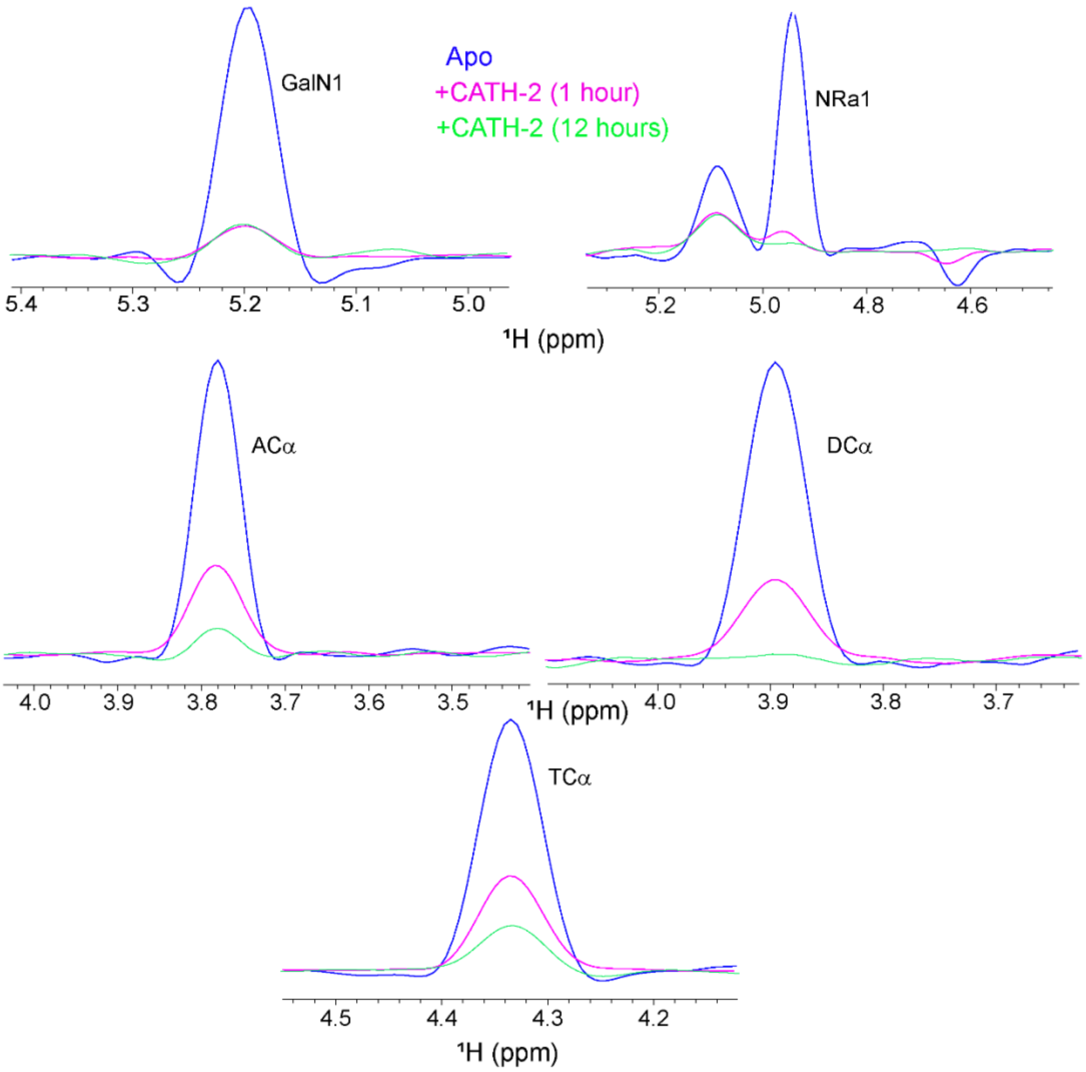

Fig. S16. Overlay of 1D slices from Fig. **2a** & Fig. **2b** showing a reduction in peak intensity.

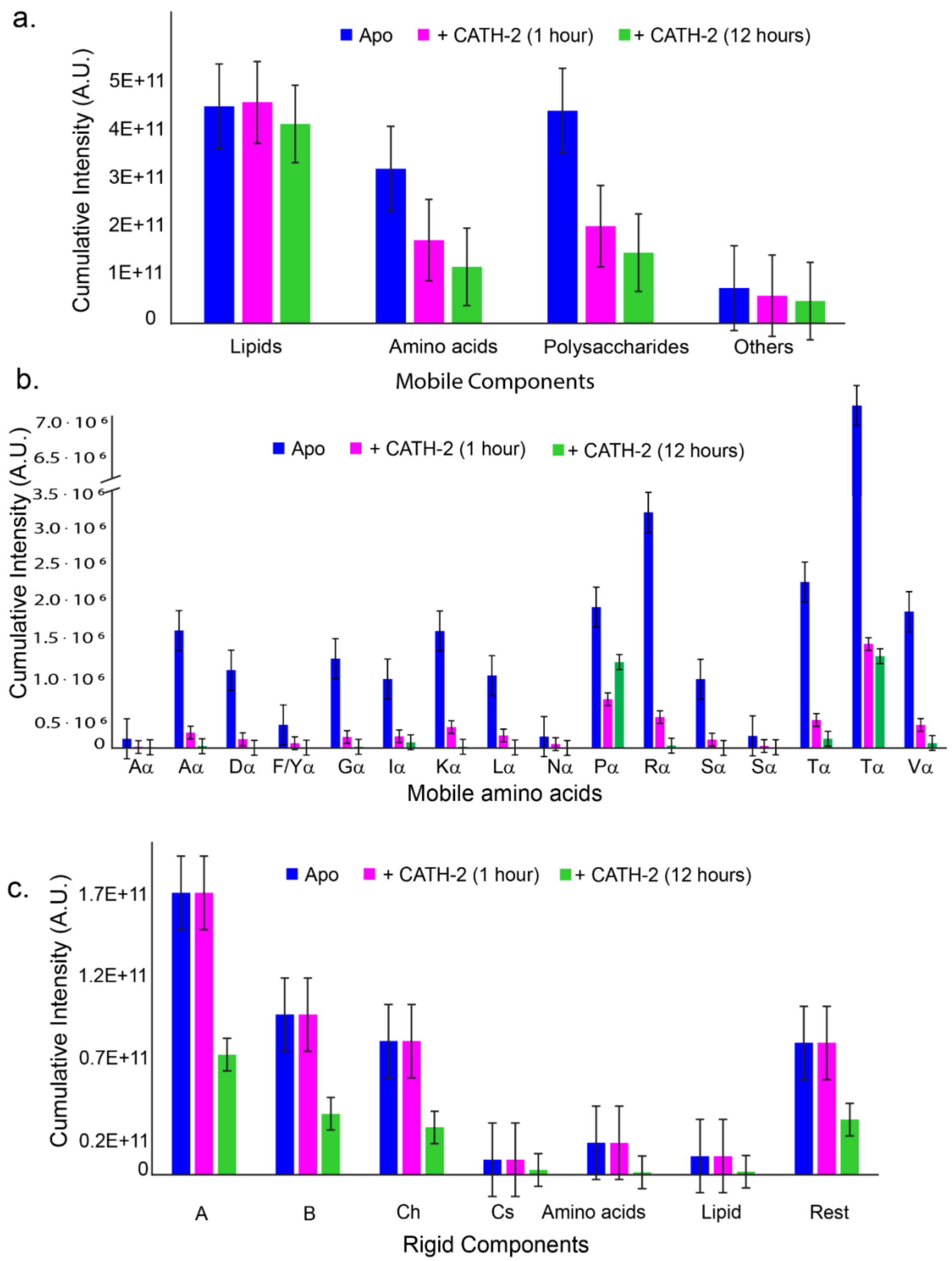

Fig. S17. (a). Cumulative intensity plot of the mobile compounds. (b). Cumulative intensity plot of the mobile amino acids (c). Cumulative intensity plot of the rigid compounds. Data are shown for apo cell walls and cell walls incubated with CATH-2 for two different exposure times.

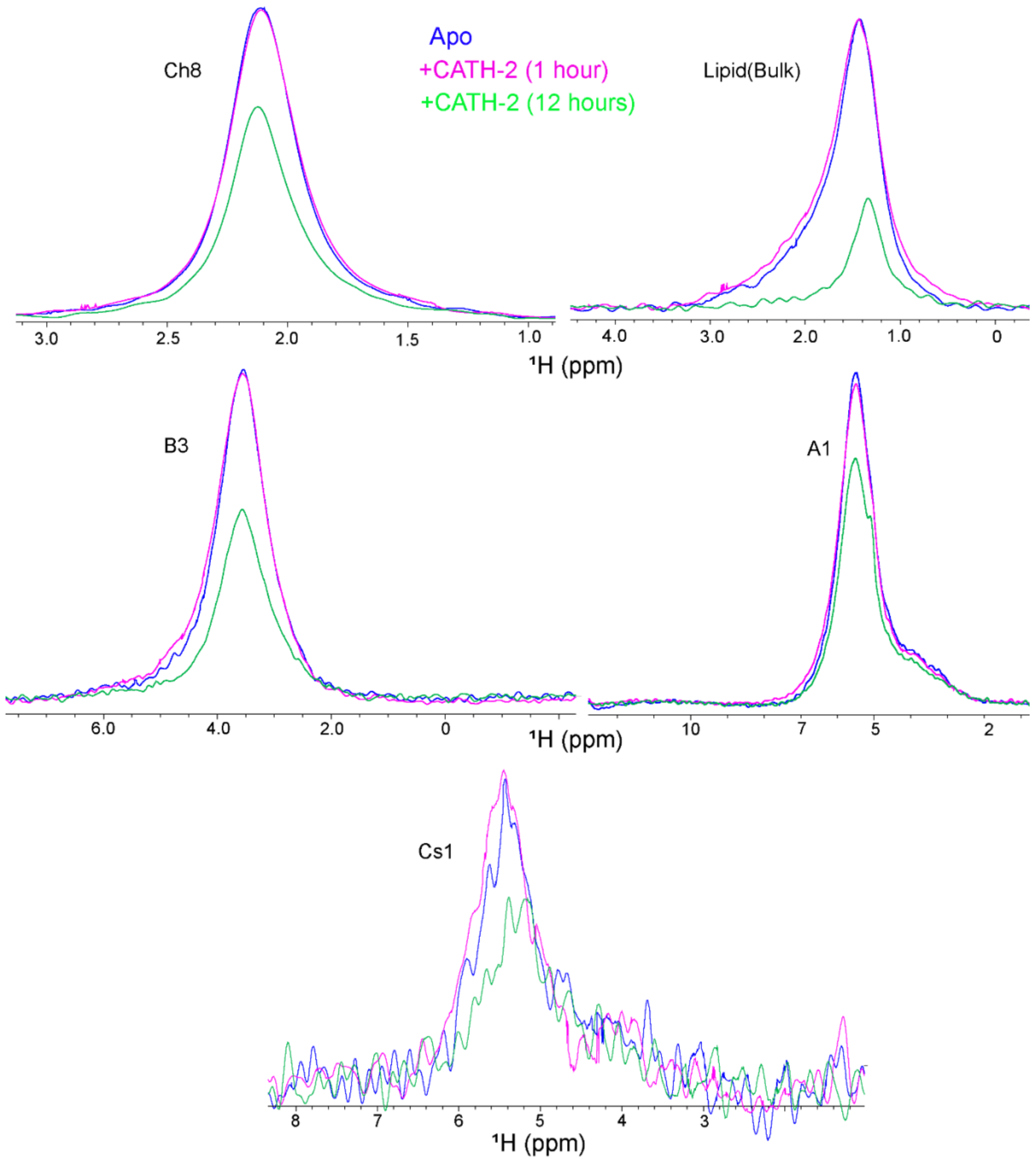

Fig. S18. Overlay of 1D slices from Fig. **3a** & **3b** showing a reduction in peak intensity.

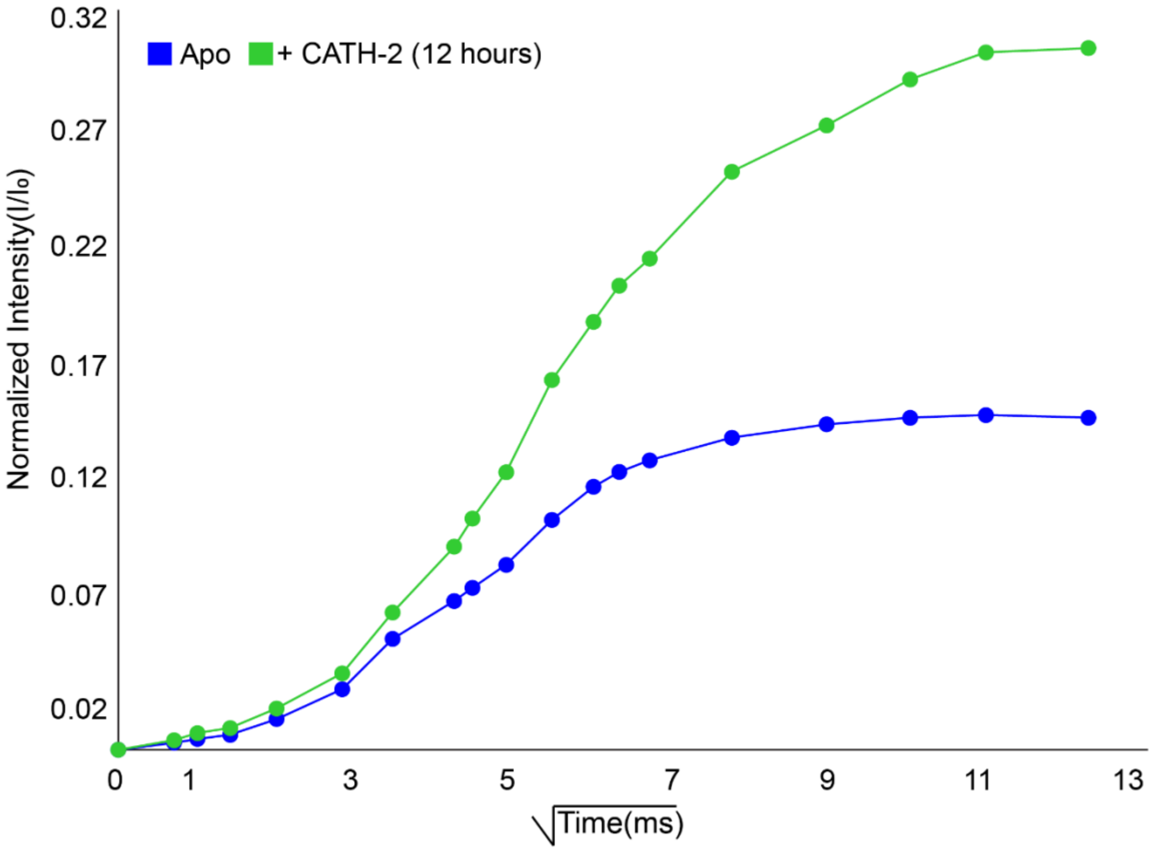

Fig. S19. Build up curves of the Apo and CATH-2 (12 hours) exposed cell walls. (I) represents the intensity of 1D hCH using different ^1H-^1H mixing times and (I_0) represents the signal intensity under equilibrium conditions (i.e., regular hCH spectrum).

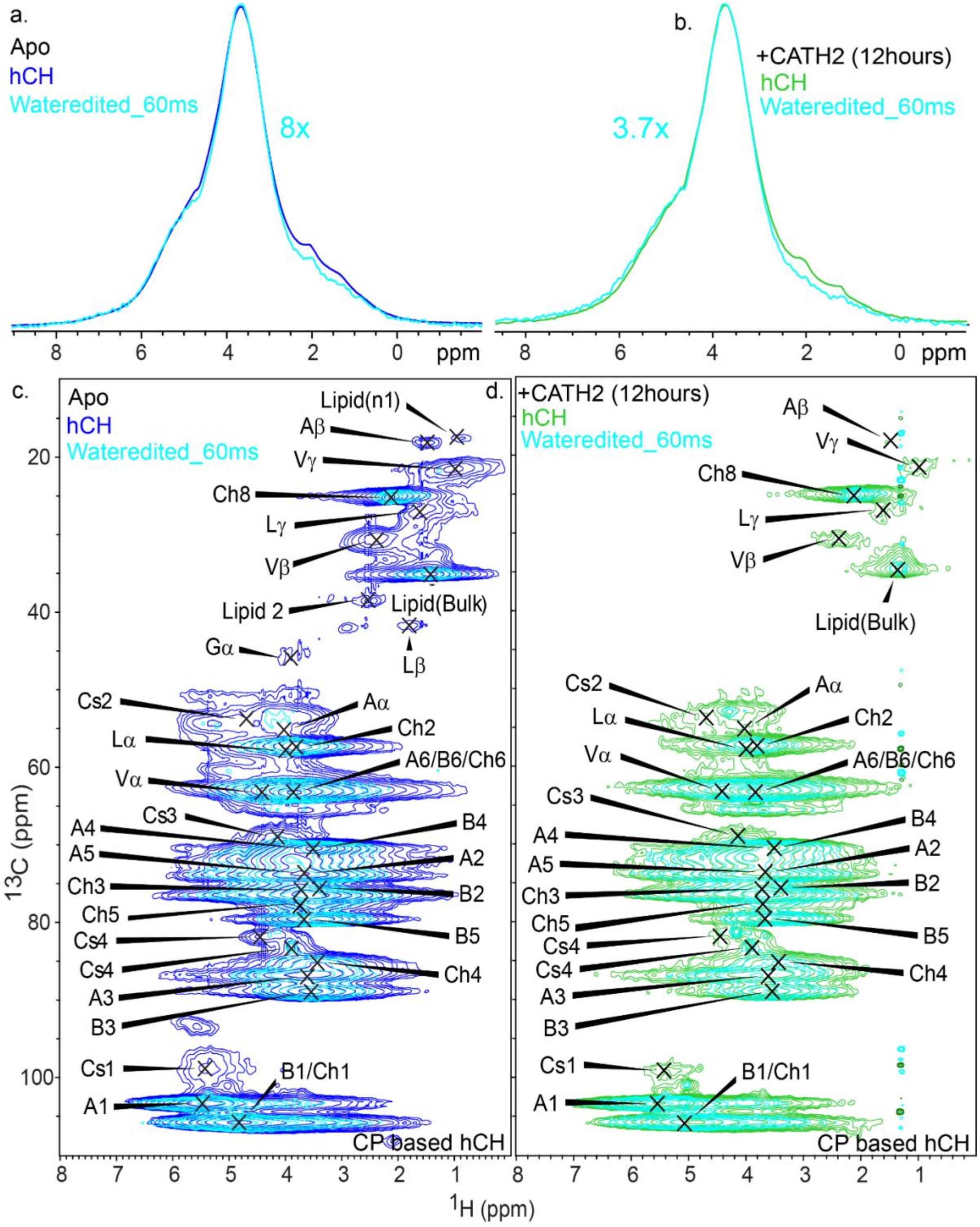

Fig. S20. Analysis of $H_2$0-edited hCH ssNMR experiments. Comparison of the 1D spectrum of the regular hCH to a water-edited hCH spectrum (60ms); (**a**). Apo and (**b**) after 12 hours treatment. (**c**) Comparison of the 2D spectrum of the regular hCH to water-edited hCH (60ms) (**c**) using cell walls without peptide treatment. (**d**) Comparison of the 2D spectrum of the regular hCH to water-edited hCH (60ms) (**c**) using cell walls after 12 hours of treatment.

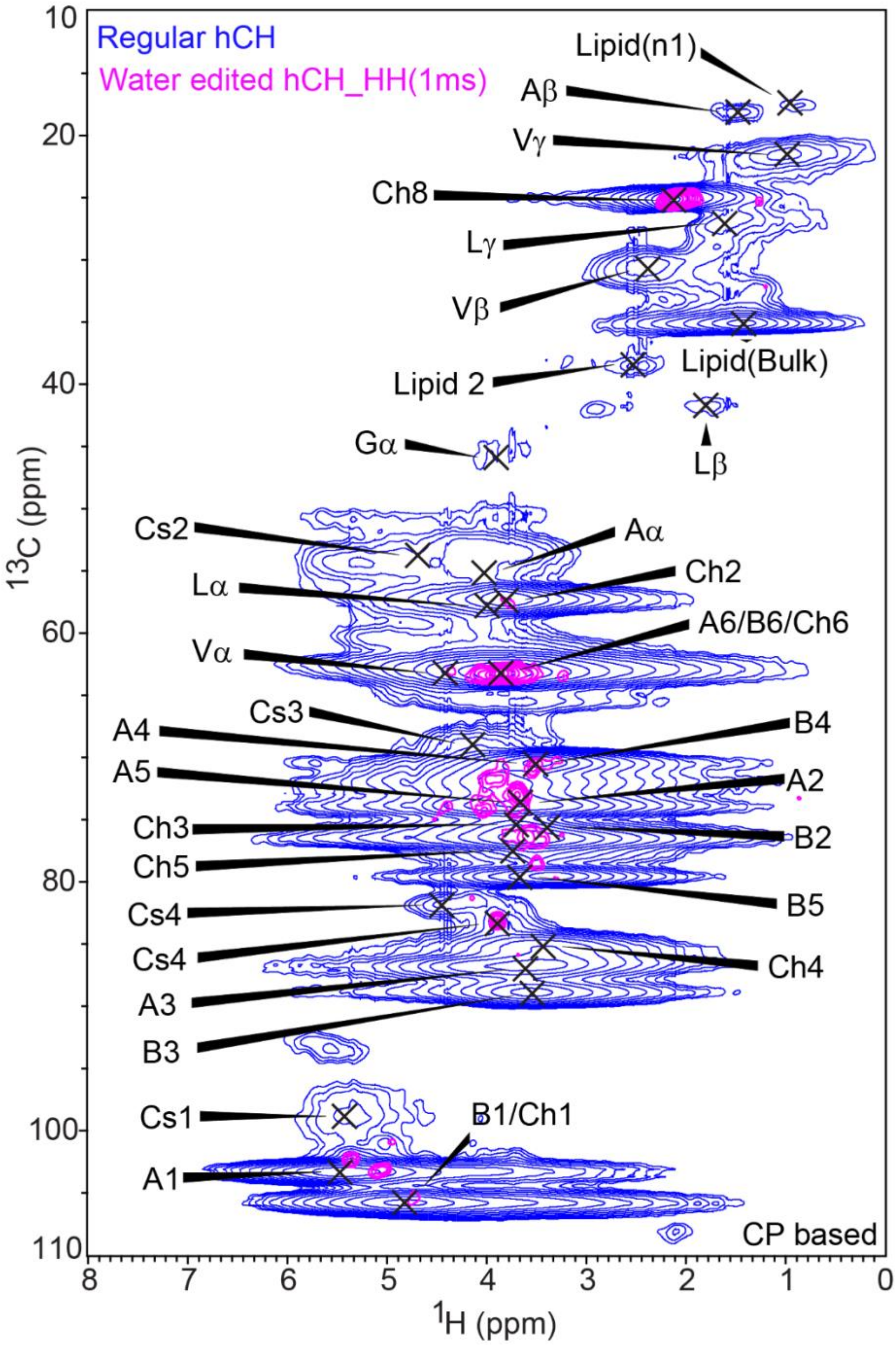

Fig. S21. Comparison of the 2D spectrum of the regular hCH to water-edited hCH (HH mixing - 1ms).

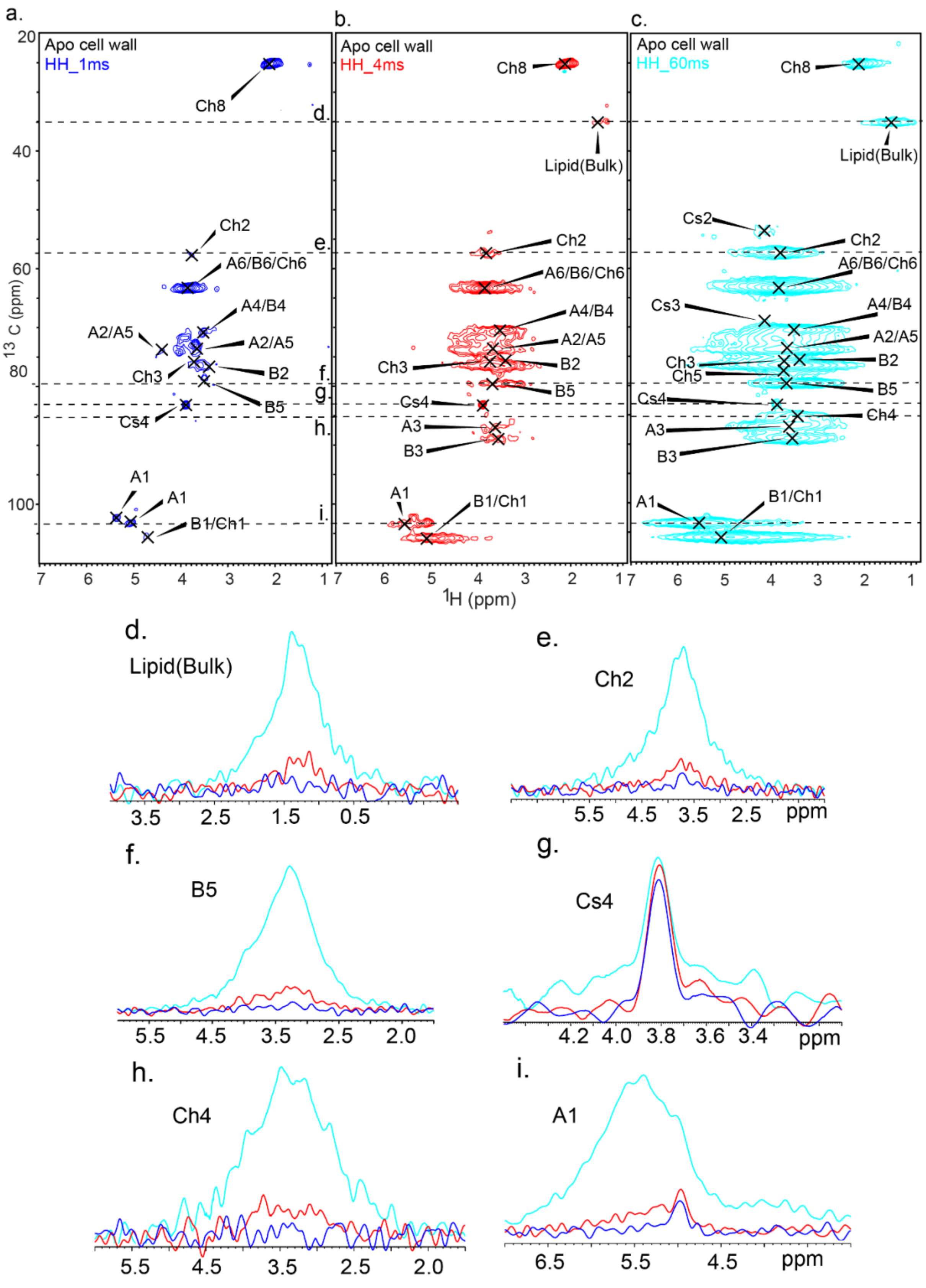

Fig. S22. Water edited 2Ds with varying (^{1}H,^{1}H) mixing time showing a gradual increase in the ^{1}H line width with increasing ^{1}H^{1}H mixing time. Data are shown in 2D (**a-c**) and for the selected 1D slices (**d-i**).

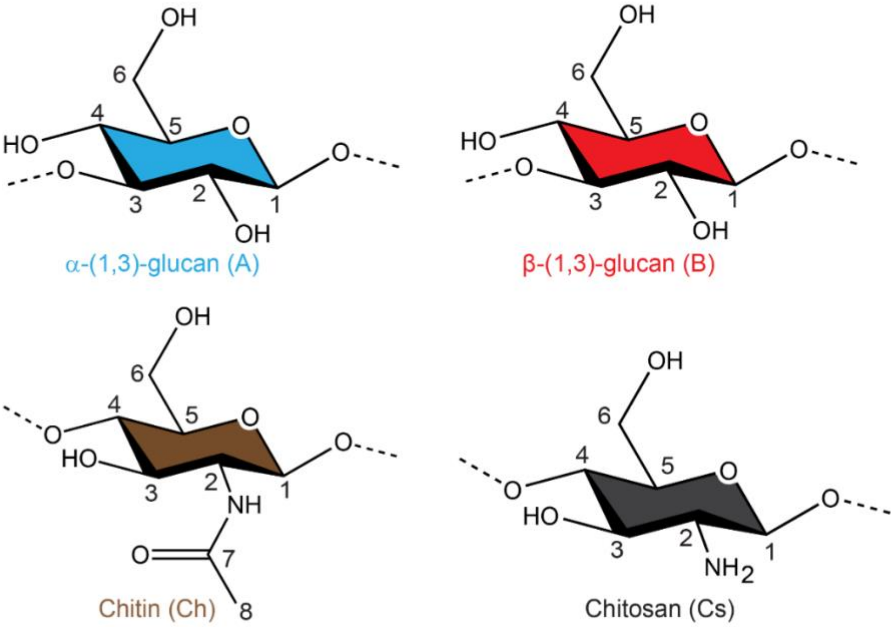

Fig. S23. Structure of the polysaccharides identified in the rigid domain.

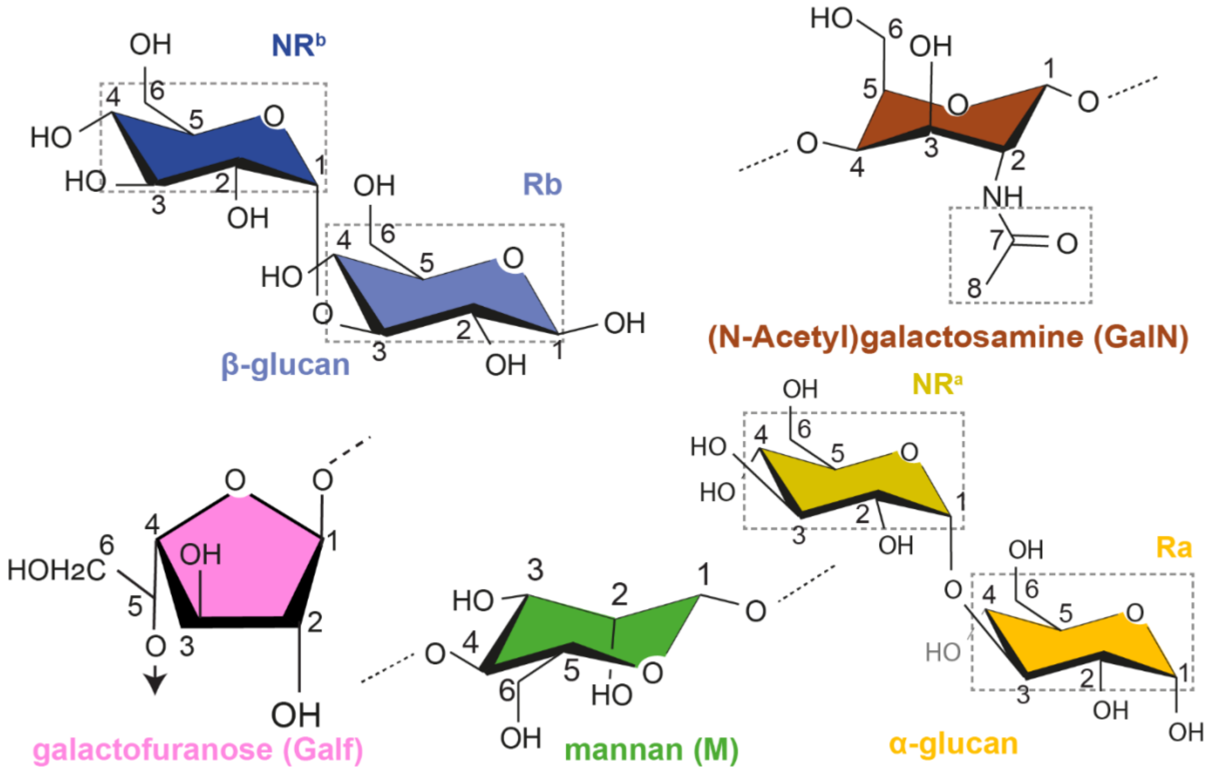

Fig. S24. Structures of the polysaccharides identified in the mobile domain.

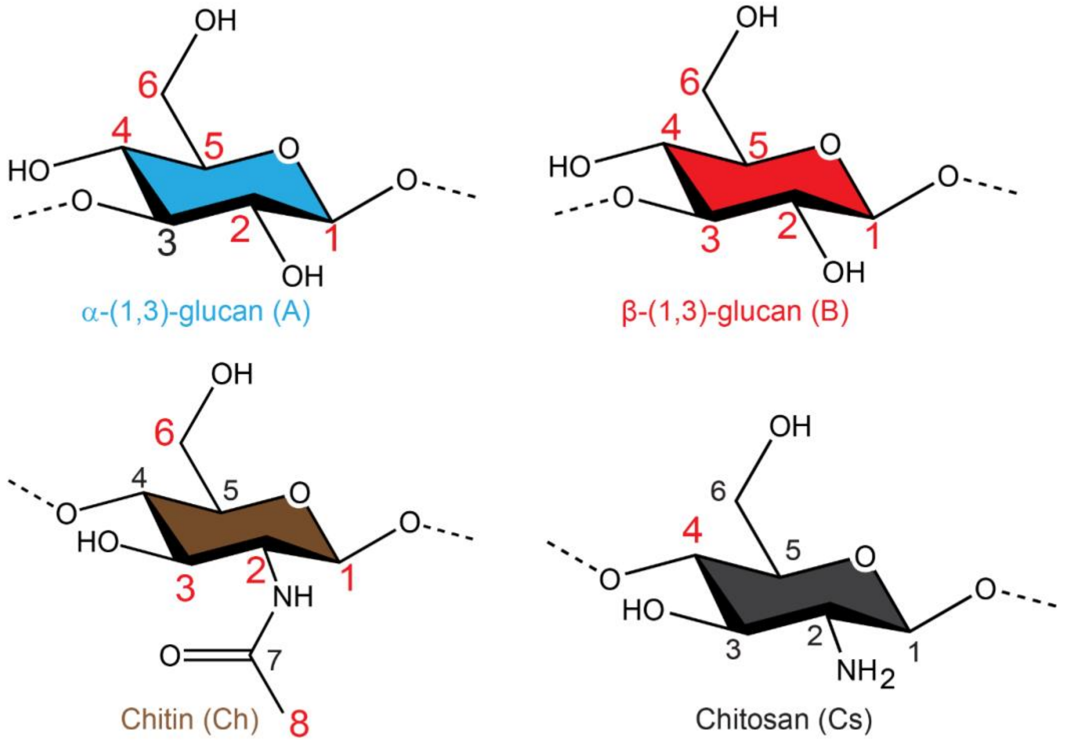

Fig. S25. Structural representation of the polysaccharides: The atoms of the monomorphic polysaccharides detected from the water edited with 1ms HH mixing time are highlighted in red, and the chemical shifts are given in the Table S8.

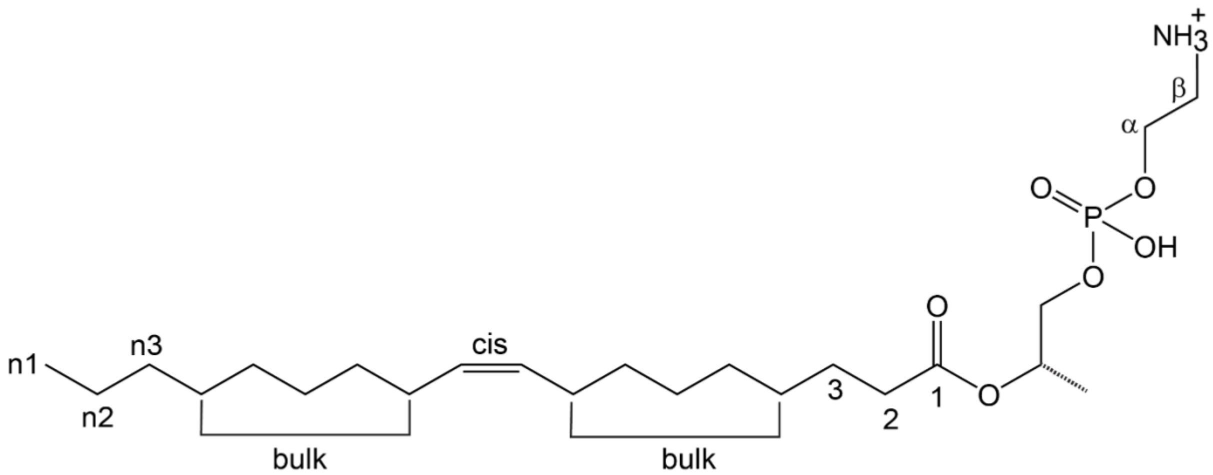

Fig. S26. Schematic representation of lipid structure.

Supporting Tables

Table S1. Acquisition parameters for the ^{1}H-detected ssNMR experiments.

Sample	Exp	NS	DS	D1 (Sec)	^{1}H 90 (kHz)	^{13}C/^{15}N 90 (kHz)	^{1}H dec (kHz)	^{13}C dec (kHz)	H_2O supp (kHz)	Contact Time (μs)	^{1}H acqu (ms)	^{13}C acqu (ms)	B_0 (MHz)	Mas (kHz)	T_{set} (K)
Apo	Scalar 2D hCH	256	32	1.4	200	91	10	10	29	-	29	10	700	60	260
Apo	Dipolar 2D hCH	128	32	1.2	200	91	15	15	29	CP:1000 BCP:200	20	8	700	60	260
Apo	Dipolar 2D hNH	256	32	1.2	200	66	15	15	29	CP:1800 BCP:600	20	10	700	60	260
+ 1hr	Scalar 2D hCH	256	32	1.4	200	91	10	10	29	-	29	10	700	60	260
+ 1hr	Dipolar 2D hCH	128	32	1.2	200	91	15	15	29	CP:1000 BCP:200	20	8	700	60	260
+ 12hr	Scalar 2D hCH	256	32	1.4	200	91	10	10	29	-	29	10	700	60	260
+ 12hr	Dipolar 2D hCH	128	32	1.2	200	91	15	15	29	CP:1000 BCP:200	20	8	700	60	260
Apo	Dipolar 2D hCCH	128	32	1.2	200	91	15	15	29	CP:1000 BCP:200 CC(8ms)	20	8	700	60	260

Table S2. Acquisition parameters for the ^{13}C-detected ssNMR experiments.

Exp	NS	DS	D1 (Sec)	^{1}H 90 (kHz)	^{13}C90 (kHz)	^{1}H dec (kHz)	PARIS[3] Mixing time(ms)	^{1}H acqu (ms)	^{13}C acqu (ms)	B_0 (MHz)	Mas (kHz)	T_{set} (K)
PDSD	64	32	2.2	67	49	80	30	10	5.6	700	15	273
^{15}N-filtered PDSD	64	32	2.2	67	49	80	30	10	5.6	700	15	273
TOBSY	64	32	2.2	67	49	10	-	23	5.6	700	13	280
PDSD	96	32	2.2	80	60	80	30	12	5.9	1200	20	245
PDSD	96	32	2.2	80	60	80	500	12	5.9	1200	20	245

Table S3. Numerical analysis of individual amino-acid occurrences present in all Rod proteins[8]. Amino acids in marked in yellow were detected in this study.

AA	RodA	RodB	RodC	RodD	RodE	RodF	RodG	Total
Leu(L)	17	17	20	17	23	15	18	127
Ala(A)	18	17	14	21	17	19	12	118
Ser(S)	11	9	16	25	7	27	11	106
Gly(G)	19	13	15	12	7	10	9	85
Val(V)	11	12	14	12	7	14	9	79
Thr(T)	6	10	6	11	16	19	10	78
Cys(C)	8	8	8	8	11	9	8	60
Asn(N)	11	10	5	11	15	4	2	58
Pro(P)	7	4	9	7	7	15	9	58
Gln(Q)	10	8	9	7	7	10	4	55
ILE(I)	12	8	8	5	4	4	6	47
Asp(D)	9	6	8	6	3	8	5	45
Lys(K)	9	7	6	5	3	8	4	42
Arg(R)	1	0	6	9	7	10	1	34
Glu(E)	3	2	3	8	2	5	9	32
Met(M)	1	1	3	5	2	7	3	22
Phe(F)	4	3	2	5	2	5	1	22
His(H)	1	3	1	5	4	0	3	17
Tyr(Y)	1	2	2	10	1	1	0	17
Trp(W)	0	0	0	3	0	0	1	4

Table S4. Chemical shifts of the polysaccharides detected in the rigid domain. The chemical shifts were obtained by the one bond correlation experiments (^{13}C- detected CP based PARIS and ^{1}H-detected CP based hCCH) and cross validated by the earlier literature values[9] and the database[10].

Sugars	C1 (ppm) H1 (ppm)	C2 (ppm) H2 (ppm)	C3 (ppm) H3 (ppm)	C4 (ppm) H4 (ppm)	C5 (ppm) H5 (ppm)	C6 (ppm) H6 (ppm)	C8 (ppm) H8 (ppm)
A	103.46, 5.50	73.85, 3.77	86.82, 3.61	70.5, 3.48	73.85, 3.77	63.35, 3.86	
B	105.9, 4.84	75.95, 3.40	89.05, 3.54	70.5, 3.48	79.58, 3.66	63.35, 3.86	
Ch	105.9, 4.84	57.63, 3.53	76.02, 3.74	85.23, 3.42	78.07, 3.70	63.35, 3.86	25.23, 2.09
Cs	99.08, 5.43	53.72, 4.68		83.13,4.15; 81.89,4.48			

List of abbreviations: A: α-(1,3)-glucan, B: β-(1,3)-glucan, C: Chitin, Cs: Chitosan

Table S5. Chemical shifts of the amino acids detected in the rigid domain. The chemical shifts were obtained by the one bond correlation experiments (^{13}C- detected CP based CC PARIS and ^{1}H-detected CP based hCCH) and cross validated by the earlier literature values[11] and BMRB amino acids database[12].

Amino acids	Cα (ppm), Hα (ppm)	Cβ (ppm), Hβ (ppm)	Cγ (ppm), Hγ (ppm)
Ala (A)	55.31, 4.02	18.14, 1.50	
Gly (G)	45.76, 3.90		
Leu (L)	57.77, 3.99	41.70, 1.80	27.21, 1.60
Val (V)	63.35, 4.37	30.64, 2.38	21.56, 1.01

Table S6. Chemical shifts of the polysaccharides detected in the mobile domain. The chemical shifts were obtained by the one-bond correlation experiments (^{13}C- detected TOBSY and ^{1}H-detected INEPT based hCCH) and cross validated by literature values[9] and the database[10].

Sugars	C1 (ppm), H1 (ppm)	C2 (ppm), H2 (ppm)	C3 (ppm), H3 (ppm)	C4 (ppm), H4 (ppm)	C5 (ppm), H5 (ppm)	C6 (ppm), H6 (ppm)
B^{a}	105.55, 4.75	76.27, 3.36	87.27, 3.76	72.88, 3.69	78.71, 3.47	
B^{b}	105.43, 4.51					
B^{c}	103.2, 4.87					
B^{d}	105.38, 4.51					
B^{e}			79.49, 4.10	70.88, 3.52	77.1, 3.63	71.6, 3.92
Rb	98.71, 4.64	77.0, 3.20	87.27, 3.76	72.4, 3.41	78.71, 3.47	
NRb	104.93, 5.08	75.71, 3.71	78.71, 3.47	72.3, 3.85	78.71, 3.47	
Ra	94.99, 5.19	74.32, 3.53	83.27, 3.89	72.4, 3.41	75.71, 3.71	
NRa	100.8, 4.95	72.2, 3.99		71.27, 3.79	69.82, 3.75	
M	102.5, 5.35	75.38, 3.52	73.04, 4.06	78.71, 3.47	69.82, 3.75	
GalN	93.71, 5.20	56.93, 3.85				
GalNAc	97.77, 4.72	59.65, 3.66				
Galf	109.77, 5.22	84.1, 4.15	79.93, 3.63			

List of abbreviations:

$B^{a,b,c,d}$: β-(1,3)-glucan
B^{e}: β-(1,6)-glucan
Rb: Reducing end β-(1,3)-glucan
NRb: Non-reducing end β-(1,3)-glucan

Ra: Reducing end α-(1,3)-glucan
NRa: Non-reducing end α-(1,3)-glucan
M: Mannan
GalN: Galactosamine
GalNAc: N-acetyl galactosamine
Galf: Galactofuranose

Underlined and double underlined chemical shifts of selected sugar resonances in Table S6 disappeared after exposure to CATH2 for 1 hour and 12 hours, respectively.

Table S7. Chemical shifts of the amino acids detected in the mobile domain. The chemical shifts were obtained by the one bond correlation experiments (^{13}C- detected TOBSY and ^{1}H-detected INEPT based hCCH) and cross validated by the earlier literature values[11] and BMRB amino acids database[12].

Amino acids	Cα (ppm),<br>Hα (ppm)	Cβ (ppm),<br>Hβ (ppm)	Cγ (ppm),<br>Hγ (ppm)	Cδ (ppm),<br>Hδ (ppm)
Ala (A)	53.49,<br>3.78	19.09,<br>1.47		
Ala (A)	52.57,<br>4.31	19.48,<br>1.39		
Arg (R)	57.52,<br>3.75	29.74,<br>2.00		
Asn (N)	54.27,<br>4.00			
Asp (D)	55.18,<br>3.89	39.37,<br>2.80		
Gly (G)	44.48,<br>3.55			
Ile (I)	62.53,<br>3.63	38.66,<br>1.96	27.39, 1.56;<br>17.47, 0.99	13.94,<br>0.92
Leu (L)	56.41,<br>3.72	42.64,<br>1.67	26.84,<br>1.70	23.77, 0.93;<br>24.85, 0.95
Phe/Tyr<br>(F/Y)	58.95,<br>3.98	39.38,<br>2.66		
Pro (P)	64.53,<br>4.06	31.85,<br>2.25	26.54,<br>1.99	
Ser (S)	58.50,<br>4.49	63.12,<br>3.96		
Ser (S)	59.34,<br>3.84	63.89,<br>3.80		
Thr (T)	62.25,<br>4.33	68.76,<br>4.25	22.32,<br>1.32	
Thr (T)	63.89,<br>3.80	69.58,<br>3.99	21.76,<br>1.20	
Val (V)	63.20,<br>3.59	31.85,<br>2.25	20.78, 1.02;<br>19.48, 0.97	

Table S8. Chemical shifts of the crystallite polysaccharides found at the water interface of the rigid domain. The chemical shifts were obtained by the one bond correlation experiment (^{1}H-detected CP based hCCH).

Species	C1 (ppm), H1 (ppm)	C2 (ppm), H2 (ppm)	C3 (ppm), H3 (ppm)	C4 (ppm), H4 (ppm)	C5 (ppm), H5 (ppm)	C6 (ppm), H6 (ppm)	C8 (ppm), H8 (ppm)
A	103.14,5.06; 102.30,5.37	73.84,4.40; 71.72,3.93; 74.26,4.02; 72.99,3.69		70.88,3.52	73.84,4.40; 71.72,3.93; 74.26,4.02; 72.99,3.69	63.35,3.86; 63.06,4.36; 63.48,3.24	
B	105.4,4.74	75.95,3.40		70.88,3.52	78.56,3.49	63.35,3.86; 63.06,4.36; 63.48,3.24	
Ch	105.4,4.74	57.63,3.53	76.02,3.74			63.35,3.86; 63.06,4.36; 63.48,3.24	25.23,2.09
Cs				83.13,3.89			

Table S9. Chemical shifts of lipid signals detected in the rigid domain. The chemical shifts were obtained by the one bond correlation experiments (^{13}C- detected CP based CC PARIS and 1H-detected CP based hCCH) and cross validated by the earlier literature values[13].

	C (ppm), H (ppm)
n1	17.44, -
C2	38.12, 2.73
bulk	35.17, 1.41

Table S10. Chemical shifts of lipid signals detected in the mobile domain. The chemical shifts were obtained by the one bond correlation experiments (^{13}C- detected TOBSY and ^{1}H-detected INEPT based hCCH) and cross validated by literature values[13].

	C (ppm), H (ppm)
n1	16.54, 0.85
n2	25.32, 1.27
n3	34.49, 1.23
C3	27.37, 1.57
bulk	32.15, 1.25
bulk	36.50, 2.22
α	65.60, 3.76

Table S11. Volumes of the defined integration areas and the relative contributions of the rigid domain components.

Integrals	Rigid components	Integral volume	Relative contribution (%)
1	Lipid(n1)	2.20×10^{8}	0.0005
2	Aβ	3.85×10^{8}	0.0008
3	Vγ	3.86×10^{9}	0.0083
4	Ch8	1.18×10^{10}	0.0254
5	Lγ	2.03×10^{9}	0.0044
6	Vβ	5.78×10^{9}	0.0124
7	Lipid(Bulk)	1.03×10^{10}	0.0223
8	Lipid(C2)	3.37×10^{8}	0.0007
9	Lβ	3.06×10^{8}	0.0007
10	Gα	5.21×10^{8}	0.0011
11	Cs2+Aα	1.03×10^{10}	0.0232
12	Ch2+Lα	2.05×10^{10}	0.0441
13	A6+B6+Ch6+Vα	7.01×10^{10}	0.1509
14	A2+A4+A5+B4	1.23×10^{11}	0.2657
15	Ch3+Ch5+B2	5.94×10^{10}	0.1279
16	B5	1.64×10^{10}	0.0352
17	Cs4	2.80×10^{9}	0.0060
18	Ch4	1.12×10^{10}	0.0240
19	A3	2.78×10^{10}	0.0598
20	B3	1.57×10^{10}	0.0338
21	Cs1	3.25×10^{9}	0.0070
22	A1	2.87×10^{10}	0.0618
23	B1+Ch1	3.90×10^{10}	0.0839
	Total	4.64×10^{11}	100

Table S12. Volumes of the defined integration areas and the relative contributions of the rigid domain components.

Rigid components	Average rel. contribution per atom (%)	contribution in the rigid domain (%)	contribution in the rigid domain (volume)
A	0.0618	$0.0618 \times 6 = 37$	1.72×10^{11}
B	0.0352	$0.0352 \times 6 = 21$	9.76×10^{10}
Ch	0.0254	$0.0254 \times 7 = 17$	7.90×10^{10}
Cs	0.0060	$0.0060 \times 3 = 2$	9.30×10^{9}
Amino acids	-	4	1.86×10^{10}
Lipids	-	2	9.30×10^{9}
Others	-	17	7.90×10^{10}
Total	-	100	4.65×10^{11}

Materials and Methods

Peptide synthesis

This study includes the use of chicken CATH-2, which was synthesized by ChinaPeptides Co., Ltd. (Chuanghong Road, Shanghai China) using classical solid-phase peptide synthesis (SPPS) and 9-fluorenylmethoxycarbonyl (Fmoc) as a protective group at the N-terminus[14]. After synthesis, the peptide was purified by HPLC with a linear gradient system (gradient: 5-95% B in 7 minutes, flow 1: ml/min, eluent A: 100% CH_3CN + 0.1% (v/v) TFA; eluent B: 100% H_2O + 0.1% TFA) using a Kromasil 100-5C18 (4.6 mm x 250 mm, 5 µm) and detection at 220 nm. All peptides used were ≥95% pure as verified by electrospray ionization Mass Spectrometry. To enhance stability and activity, the peptide was amidated at their C-terminus ($-NH_2$).

Sample preparation for the Solid-State NMR spectroscopy

A. fumigatus (strain Af293.1, Prg3AMA1-RFP) was grown for three days at 37°C on potato dextrose agar (PDA)[15]. Conidia were harvested with 0.9 % (w/v) NaCl and filtered through fiberglass to remove remnants of mycelium and hyphae. Conidia were counted using a Bürker Türk counting chamber. For the Solid-State NMR sample preparation, 100 ml NMR medium (20 g/L glucose, 1 g/L NH_4Cl) was grown at 37°C at 250 rpm for 5 days, inoculated with 10^5 spores. The mycelium was harvested and divided into three parts. One part of them was incubated with 0.2 % formaldehyde for 1 hour at 37°C and lyophilized overnight, which was named as apo (untreated) cell wall. Other two parts were exposed to CATH-2 for 1 hour and 12 hours, which are referred to as short and long term exposure, respectively. The CATH-2 samples were incubated with 0.2 % formaldehyde for 1 hour at 37°C and lyophilized overnight as the apo cell wall. The lyophilized materials were then added to separate 1.5 mL Eppendorf tubes with metal beads and homogenized using the TissueLyser III (Qiagen). All the three samples were washed 4 times with MQ, each time followed by centrifugation at 6500 rpm for 10 minutes.

Germination kinetics with oCelloScope

96 wells suspension culture plates (Greiner bio-one, Cellstar 655185) were used for the germination kinetics experiment. Media consisted of minimal medium (MM: 70.6 mM NaNO3, 11 mM KH2PO4, 6.7 mM KCl, 2 mM MgSO4•7H2O, and tracer elements in accordance with Vishniac and Santer (1957)), different concentrations (0.1, 0.5, 1 and 5 µM) of AMPs and 10 µM L-alanine were added. For the positive germination control only 10 µM L-alanine was added and for the negative control this was substituted with MQ. 4000 *A. fumigatus* (Af293.1) spores were added per well. Every condition contained three technical replicates. The oCelloScope was placed inside an incubator set at 37°C. The plate was placed in the oCelloScope for one hour before the measurements started to ensure that the spores settled on the bottom. The oCelloScope took one picture every hour for 24 hours. A minimum of 10 pixels and a maximum of 700 pixels was used. Uniexplorer version 8.1.0.7682 (RL2) was used as the software to control the oCelloScope. Area, circularity and X/Y position were determined by the software.

Solution-State NMR spectroscopy experiments

The CATH-2 peptide was dissolved in MQ water (10% D_2O). One-dimensional ^1H (Proton) solution-state NMR experiments were carried out on 600 MHz Bruker Avance III spectrometer equipped with a triple resonance cryogenic-probe at 298K. Spectra were processed and analysed with Bruker Topspin 3.6.2 software. Proton chemical shifts was referenced using the water resonance at 4.7 ppm.

Solid-State NMR spectroscopy experiments

For our comparative ssNMR analysis of the treated and untreated cell walls, samples were spun down at 100,000Xg at 4^0C for 1 hour to remove excessive water and packed into 1.3mm MAS (Magic angle spinning) rotors (2 mg) for the ^1H-detected experiments and 3.2mm MAS rotor (25 mg) for the ^{13}C-detected experiments, respectively. All dipolar and scalar based ^1H-detected experiments were carried out on a narrow bore 700MHz (16.5T) spectrometer at 60kHz at 260K (T_{set}) using a 1.3mm HXY MAS probe (Bruker Biospin). Because of frictional heating, the actual sample temperature was 293K, calibrated according to KBr powder sample[16]. PISSARO[17] and WALTZ 16[18] decoupling pulse sequences were used for the dipolar and scalar based experiments, respectively. The MISSISSIPI[19] pulse sequence was used for the water suppression. All the dipolar and scalar based ^{13}C-detected experiments were carried out both on narrow bore 700MHz (16.5T) and 1200MHz (28.2) spectrometers at 13-20 kHz MAS at 245K-280K T_{set} using 3.2mm Efree HXY MAS probe (Bruker Biospin). Spinal 64[20] and WALTZ 16[18] decoupling pulse sequences were used for the dipolar and scalar based experiments, respectively. All ^1H-detected and ^{13}C-detected acquisition parameters are summarized in tables **S1** and **S2,** respectively. The spectra

were processed using the Bruker Topspin 3.6.2 software. ^{1}H, ^{13}C, ^{15}N chemical shifts were referenced by H_2O, adamantane and histidine, respectively, and analysed using NMRFAM-Sparky[21].

Secondary chemical-shift analysis:

We used the equation below to analyze the secondary structure of the amino acids found in both the rigid and mobile domain using a previously described approach by Luca et al[22]:

$\Delta\delta = \delta C\alpha - \delta C\beta = \{\delta C\alpha\ (obs) - \delta C\alpha\ (\alpha h/\beta s/rc)\} - \{\delta C\beta\ (obs) - \delta C\beta\ (\alpha h/\beta s/rc)\}$

where:

$\Delta\delta$ = Difference between the experimentally observed Cα and Cβ chemical shifts and predicted isotropic chemical shifts for: αh = α-helix, βs = β-strands and rc = random coil. The isotropic chemical shifts for α-helix, β-strands and random coil were taken from Wang and Jardetzky[11].

Relative abundance analysis of CP based ^{1}H detected hCH spectra:

For a relative quantification of our ^{1}H-detected 2D ssNMR data, we made use of a peak integration method previously demonstrated to quantify the various components of fungal cell walls[9a]. Briefly, by assuming that the contribution for every CH peak of a polysaccharide species in the spectrum is equivalent, a rough estimation of the relative contribution per polysaccharides in the rigid domain was calculated. The resulting integrated peak volumes of the spectrum (in Fig. **3.2b**) are reported in table **S11**. By determining the total volume of all peaks of each component (i.e., polysaccharides/amino acids/lipids), relative contributions in the rigid domain were determined and are reported in table **S12**.

References

[1] J. Jumper, R. Evans, A. Pritzel, T. Green, M. Figurnov, O. Ronneberger, K. Tunyasuvunakool, R. Bates, A. Zidek, A. Potapenko, A. Bridgland, C. Meyer, S. A. A. Kohl, A. J. Ballard, A. Cowie, B. Romera-Paredes, S. Nikolov, R. Jain, J. Adler, T. Back, S. Petersen, D. Reiman, E. Clancy, M. Zielinski, M. Steinegger, M. Pacholska, T. Berghammer, S. Bodenstein, D. Silver, O. Vinyals, A. W. Senior, K. Kavukcuoglu, P. Kohli, D. Hassabis, *Nature* **2021**, *596*, 583-589.

[2] Y. Xiao, A. I. Herrera, Y. R. Bommineni, J. L. Soulages, O. Prakash, G. Zhang, *J Innate Immun* **2009**, *1*, 268-280.

[3] M. Weingarth, D. E. Demco, G. Bodenhausen, P. Tekely, *Chemical Physics Letters* **2009**, *469*, 342-348.

[4] A. Adler, M. Bangera, J. W. Beugelink, S. Bahri, H. van Ingen, C. A. Moores, M. Baldus, *Nature Communications* **2024**, *15*, 1948.

[5] P. Fricke, V. Chevelkov, M. Zinke, K. Giller, S. Becker, A. Lange, *Nat Protoc* **2017**, *12*, 764-782.

[6] M. Baldus, B. H. Meier, *Journal of Magnetic Resonance, Series A* **1996**, *121*, 65-69.

[7] S. Bahri, A. Safeer, A. Adler, H. Smedes, H. van Ingen, M. Baldus, *J Biomol NMR* **2023**, *77*, 111-119.

[8] I. Valsecchi, V. Dupres, E. Stephen-Victor, J. I. Guijarro, J. Gibbons, R. Beau, J. Bayry, J. Y. Coppee, F. Lafont, J. P. Latge, A. Beauvais, *J Fungi (Basel)* **2017**, *4*.

[9] aA. Safeer, F. Kleijburg, S. Bahri, D. Beriashvili, E. J. A. Veldhuizen, J. van Neer, M. Tegelaar, H. de Cock, H. A. B. Wösten, M. Baldus, *Chemistry – A European Journal* **2023**, *29*, e202202616; bX. Kang, A. Kirui, A. Muszyński, M. C. D. Widanage, A. Chen, P. Azadi, P. Wang, F. Mentink-Vigier, T. Wang, *Nature Communications* **2018**, *9*, 2747; cA. Chakraborty, L. D. Fernando, W. Fang, M. C. Dickwella Widanage, P. Wei, C. Jin, T. Fontaine, J.-P. Latgé, T. Wang, *Nature Communications* **2021**, *12*, 6346; dI. Gautam, J. R. Yarava, Y. Xu, R. Li, F. J. Scott, F. Mentink-Vigier, M. Momany, J.-P. Latgé, T. Wang, *Carbohydrate Polymers* **2025**, *348*.

[10] X. Kang, W. Zhao, M. C. Dickwella Widanage, A. Kirui, U. Ozdenvar, T. Wang, *J Biomol NMR* **2020**, *74*, 239-245.

[11] Y. Wang, O. Jardetzky, *Protein Sci* **2002**, *11*, 852-861.
[12] J. L. Markley, E. L. Ulrich, H. M. Berman, K. Henrick, H. Nakamura, H. Akutsu, *J Biomol NMR* **2008**, *40*, 153-155.
[13] R. A. M. van Beekveld, M. G. N. Derks, R. Kumar, L. Smid, T. Maass, J. Medeiros-Silva, E. Breukink, M. Weingarth, *Chemistry* **2022**, *28*, e202202472.
[14] A. van Dijk, E. M. Molhoek, E. J. A. Veldhuizen, J. L. M. T.-v. Bokhoven, E. Wagendorp, F. Bikker, H. P. Haagsman, *Molecular Immunology* **2009**, *46*, 2465-2473.
[15] S. M. Leal, Jr., S. Cowden, Y. C. Hsia, M. A. Ghannoum, M. Momany, E. Pearlman, *PLoS Pathog* **2010**, *6*, e1000976.
[16] K. R. Thurber, R. Tycko, *J Magn Reson* **2009**, *196*, 84-87.
[17] M. Weingarth, P. Tekely, G. Bodenhausen, *Chemical Physics Letters* **2008**, *466*, 247-251.
[18] A. J. Shaka, J. Keeler, T. Frenkiel, R. Freeman, *Journal of Magnetic Resonance (1969)* **1983**, *52*, 335-338.
[19] D. H. Zhou, C. M. Rienstra, *J Magn Reson* **2008**, *192*, 167-172.
[20] B. M. Fung, A. K. Khitrin, K. Ermolaev, *Journal of Magnetic Resonance* **2000**, *142*, 97-101.
[21] W. Lee, M. Tonelli, J. L. Markley, *Bioinformatics* **2015**, *31*, 1325-1327.
[22] S. Luca, D. V. Filippov, J. H. van Boom, H. Oschkinat, H. J. de Groot, M. Baldus, *J Biomol NMR* **2001**, *20*, 325-331.
